# Secondary School Teachers’ Disorder-Specific Mental Health Literacy About Depression, Anxiety, Early Psychosis and Suicide Risk: A Scoping Review

**DOI:** 10.3390/bs16010115

**Published:** 2026-01-14

**Authors:** Siann Bowman, Carol McKinstry, Linsey Howie

**Affiliations:** 1 Department of Community and Clinical Health, School of Allied Health, Human Services & Sport La Trobe University, Melbourne, VIC 3086, Australia; 2 La Trobe Rural Health School, La Trobe University, P.O. Box 199, Bendigo, VIC 3552, Australia

**Keywords:** students, teachers, mental health literacy, literacy training programs, depression, anxiety, early psychosis, suicide risk, secondary schools

## Abstract

Considering the high prevalence of adolescent depression and anxiety, the profound functional consequences of untreated early psychosis and suicide being the number one cause of death in Australia among 15–19-year-olds, ensuring that teachers are literate about these disorders should be a high priority. Teachers’ disorder-specific literacy is a pragmatic response to healthcare system constraints. This scoping review aimed to map the evidence of teacher mental health literacy training programs, specifically for depression, anxiety, early psychosis and suicide risk. PRISMA-ScR guidelines were followed. Included studies were published in English between 2000 and 2024, focused on teachers working with students in Year 7–12 and measured teachers’ knowledge of depression, anxiety, psychosis or suicide risk. Studies were appraised for quality. Eighteen studies met the inclusion criteria. Nine evaluated knowledge of student depression, five evaluated knowledge of anxiety and five evaluated knowledge of psychosis, while nine studies focused on suicide risk. Providing disorder-specific training and evaluation, rather than general mental health literacy training, is recommended for future research. When healthcare systems lack the capacity to provide care for ill adolescents, schools often function as frontline sites for recognition and triage. Disorder-specific literacy is recommended for teachers so they can manage their real-world, health-system compensation role.

## 1. Introduction

Adolescents are highly susceptible to the onset of mental ill health which can be associated with academic underachievement and withdrawal from school (McGorry et al., 2022; Moon et al., 2017; Papandrea and Winefield, 2011). Approximately 50% of all mental health conditions emerge by the age of 14 (WHO, 2023). The most common mental health conditions that emerge during adolescence are anxiety and depression (Kessler et al., 2007; Lawrence et al., 2015).

### 1.1. Adolescent Anxiety and Depression

The leading contributors to the burden of disease for young people are depression and anxiety (AIHW, 2024). Depression is defined as persistent feelings of sadness, hopelessness and loss of interest in activities once enjoyed (American Psychiatric Association, 2013). Anxiety is characterized by excessive fear and worry, which can reduce a young person’s ability to function in his or her daily life (American Psychiatric Association, 2013).

Ten years ago in Australia, depression and anxiety were found to affect approximately 14% of young people aged 12–17 (Lawrence et al., 2015). In a recent Australian longitudinal study, almost three-quarters of adolescents in the sample (*n* = 1239) experienced clinical depression or anxiety symptoms, with 72% experiencing chronic symptoms (Robson et al., 2024). This study involved tracking children’s symptoms of anxiety and depression yearly, from age 10 to age 18 (Robson et al., 2024). Depressive symptoms were measured using the 13-item Short Mood and Feelings Questionnaire (SMFQ), with a threshold score of 12 or higher used to identify depressive symptoms. Anxiety symptoms were measured using an 8-item shortened version of the Spence Children’s Anxiety Scale (SCAS), with a score of 11 or higher used to identify anxiety symptoms. The researchers found that 84 percent of girls and 61 percent of boys had depression or anxiety symptoms at least once during adolescence (Robson et al., 2024). The onset of anxiety and depression symptoms occurred at times of school stress, such as during the transition from primary to secondary school, around exams and around the completion of their school education (Robson et al., 2024).

Adolescent depression and anxiety can negatively impact the secondary school experience through poor grades and school dropout (Bowman et al., 2020; Elmelid et al., 2015). The symptoms of depression, including impaired concentration, loss of interest, low self-esteem, hopelessness and social withdrawal significantly reduce school performance (Esch et al., 2014; Frojd et al., 2008; Garvik et al., 2014). Frojd and colleagues (2008) found that depressed students focused on depressive thoughts instead of learning tasks. Anxiety disorders can also result in academic underachievement (Esch et al., 2014; Van Ameringen et al., 2003; Weldman et al., 2015). Data from the Australian National Survey of Mental Health and Wellbeing (*n* = 2055) showed depression and anxiety were significantly associated with difficulties completing Year 10 and school dropout (Leach and Butterworth, 2012).

### 1.2. Adolescent Early Psychosis

‘Early psychosis,’ or first episode psychosis (FEP), refers to the early course of a psychotic disorder that includes the prodrome and period up to five years from first entry into treatment (Early Psychosis Guidelines Writing Group, 2016). Psychotic disorders such as schizophrenia are a contributor to the burden of disease in young Australians aged 15 to 24 (AIHW, 2024). Early psychosis typically emerges in adolescence or early adulthood (Staines et al., 2023; Williams et al., 2024). An Australian study found that the incidence of treated early psychosis for adolescents aged 15 to 24 in a specific catchment area was 123.2 per 100,000, which is approximately 0.12% per year (Eaton et al., 2019). A recent UK research review reported a rate of 105.34 cases per 100,000 for young people aged 15–24 (Kelleher, 2025).

The Second Australian National Survey of Psychosis (Waghorn et al., 2012) found that only 31.9% of individuals who experienced psychosis had completed school (68.1% had not) (Waghorn et al., 2012). Other studies have found that between 44% and 58% of young people experiencing early psychosis do not finish school (Bowman et al., 2014; Goulding et al., 2010). Despite therapeutic advances over the past half-century, up to a third of those who experience early psychosis may recover, and approximately a quarter develop persisting symptoms with high levels of impairment and healthcare needs (Lally et al., 2017; Siskind et al., 2022).

Anxiety, depression and early psychosis during adolescence can have serious ramifications for individuals’ educational goals (Bernal-Morales et al., 2015; Bowman et al., 2020; OECD, 2012). Adolescents with these conditions are also more likely to engage in self-harming or suicidal behaviors (Daraganova, 2017). Young people can have difficulty recognizing their mental ill health and therefore may not seek professional help (Jorm, 2012; Radez et al., 2021). Adult assistance may be needed to recognize early symptoms (Jorm, 2012; Kelly, 2011).

### 1.3. Adolescent Suicide Risk

Globally, suicide is the third leading cause of death among 15–19-year-olds (WHO, 2020) and is the leading cause of death among young Australians aged 15–24 years (AIHW, 2025). Deaths by suicide represented 31.8% of all deaths in adolescents aged 15–17 years (AIHW, 2025). The number of deaths has increased by 15% since 2001 (AIHW, 2025). Suicidal ideation is defined as having thoughts, ideas or ruminations about dying by suicide (American Psychiatric Association, 2013). Early identification of suicidal adolescents is the key to suicide prevention. As adults who may spend a lot of time with adolescents, teachers could play a role in identifying and supporting suicidal students if they have the knowledge, confidence and skills (Productivity Commission, 2020).

There is, therefore, an increasing need for teachers to receive training to enhance their mental health literacy about student depression, anxiety, early psychosis and suicide risk. In the context of teacher training, the behavior change framework proposes that increasing teachers’ knowledge can lead to more frequent helping behavior for students in need (Kelly, 2011). Feeling confident and capable are strong predictors of helping behaviors (Rossetto et al., 2016).

### 1.4. Teachers’ Disorder-Specific Mental Health Literacy

Teachers’ mental health literacy has been gaining global recognition as a strategy to address adolescent mental health (Hugh-Jones et al., 2022; Nalipay and Simon, 2023).

Disorder-specific mental health literacy is situated within a broader systems-level context. In many educational settings, limited access to timely specialist mental health services exists. This is due to workforce shortages, long wait times or fragmented referral pathways and means that schools are increasingly expected to shoulder responsibilities related to early identification, initial response and referral decision-making. Teachers’ disorder-specific literacy is not only educationally relevant but also a pragmatic response to healthcare system constraints.

Mental health literacy is defined as “knowledge and beliefs about mental health problems which aid their recognition, management or prevention” (Jorm et al., 1997, p. 184). This definition acknowledges the need to recognize specific illnesses, understand the treatment options, have a positive attitude towards recognition and implement support to those experiencing mental ill health (Jorm, 2012; Jorm et al., 1997). Without mental health literacy, teachers are at risk for both under-identification and over-interpretation of normal life problems (Foulkes and Andrews, 2023; Radez et al., 2021). Teachers with good mental health literacy may be able to recognize depression, anxiety, early psychosis and suicide risk in students, refer them for early intervention and feel more confident managing students with mental ill health in class (Miller et al., 2019).

Several recent systematic reviews have investigated the mental health literacy programs for teachers but have not specifically focused on teachers’ knowledge of depression, anxiety or early psychosis. Anderson et al. (2019) reviewed mental health literacy programs for teachers in middle and senior schools and included eight studies from Australia, the UK, Haiti, Canada and Malawi. The review did not focus specifically on teachers’ knowledge of depression, anxiety, early psychosis or suicide risk, but instead focused on knowledge of “common adolescent mental health issues” (p. 504), behaviors and attitudes aimed towards helping students. Training programs for secondary school teachers were found to be effective at improving knowledge about mental health but not at increasing helping behaviors. The review by Yamaguchi et al. (2020) examined sixteen studies about general mental health literacy programs for primary and secondary school teachers. Studies were conducted in Australia, Brazil, Canada, Chile, Germany, Malawi, Pakistan, Tanzania, the UK and the USA. The authors recommended that better evidence was needed before the effectiveness of programs could be established. In another review, Ohrt et al. (2020) examined fifteen studies focusing on mental health literacy programs for both primary and secondary school teachers. The studies were conducted in a range of countries, and only eight studies demonstrated improvements in general mental health literacy and a reduction in stigmatizing attitudes.

In 2024, Prabhu and colleagues conducted a systematic review and narrative synthesis to identify what was known globally about the interventions to improve secondary school teachers’ mental health literacy. The aim was to provide an update on the evidence to inform program commissioning and identify knowledge gaps. They included twenty studies from both high-income countries (eleven studies) and low- to middle-income countries (thirteen studies). They found that interventions to improve mental health literacy can be effective in the short term; however, the quality of the evidence needs to improve to better inform practice. The authors identified that a limitation of their review was not including studies about disorder-specific literacy. The current scoping review builds on Prabhu and colleagues’ work (2024) by addressing this gap, specifically, teachers’ mental health literacy about depression, anxiety, early psychosis and suicide risk.

### 1.5. Aims

This current review addresses the gap identified in previous studies by focusing on disorder-specific mental health literacy of teachers. The aim of this scoping review was to describe and report on the effectiveness of mental health literacy training programs for secondary school teachers relating to depression, anxiety, early psychosis or suicide risk.

A scoping review was chosen over a systematic review because the purpose was to scope the body of literature, clarify concepts and investigate the way research has been conducted. It also sought to identify key characteristics of teachers’ disorder-specific mental health literacy (depression, anxiety, early psychosis and suicide risk) and identify knowledge gaps. This scoping review used rigorous and transparent methods to ensure that the results were trustworthy; therefore, the review followed the methodological framework in accordance with Arksey and O’Malley’s (2005), Levac et al.’s (2010) and Peters et al. (2021).

Two purpose statements for this review were developed: (a) identify and summarize the literature that explores the disorder-specific mental health literacy of secondary school teachers, specifically about student depression, anxiety, early psychosis and suicide risk; and (b) describe and analyze the effectiveness of the disorder-specific mental health literacy programs. The research question formulated was “What is the research evidence relating to the disorder-specific mental health literacy of secondary school teachers, specifically depression, anxiety, early psychosis and suicide risk?”

## 2. Methods

The Preferred Reporting Items for Systematic reviews and Meta-Analyses extension for Scoping Review guidelines were followed (PRISMA-ScR) (Tricco et al., 2018). The initial search began in June 2023 and was updated in December 2024. Key search terms included “mental illness,” “depression,” “anxiety,” “early psychosis,” “suicide,” “adolescent,” “student,” “pupil,” “youth,” AND “school-based,” “high school,” “secondary school,” “teacher,” “education*” OR “mental health literacy” OR “literacy” OR “mental health knowledge” “education program” OR “health education” OR “intervention” OR “education intervention” OR “school-based” OR gatekeep* (gatekeeper, gatekeeping). In research, mental health literacy and disorder-specific knowledge are operationalised as measurable constructs to assess an individual’s ability to recognize, manage and prevent mental health conditions (Worley et al., 2025). Disorder-specific knowledge is a construct focusing on the depth of understanding regarding a single, specific condition rather than mental health in general.

**Figure 1:**
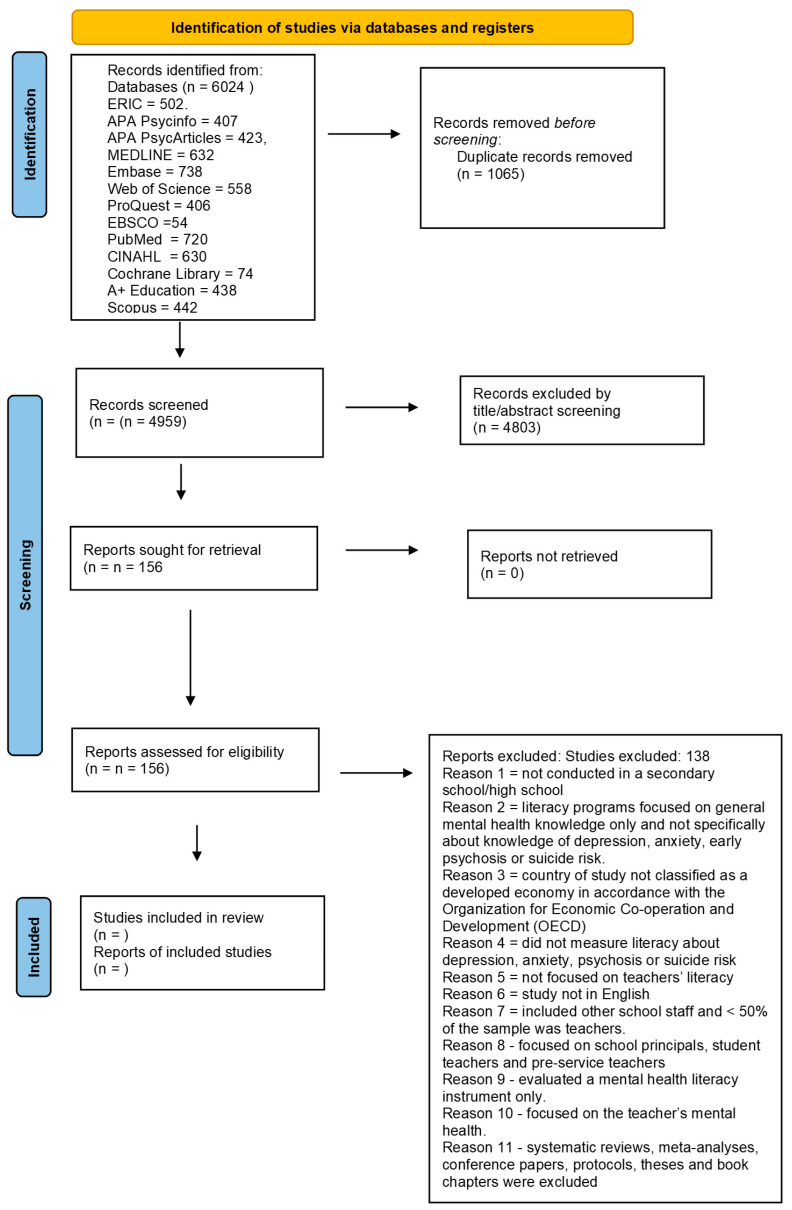
Secondary school teachers’ disorder-specific mental health literacy.

Searches were completed in thirteen databases, including ERIC, APA Psycinfo, APA PsycArticles, MEDLINE, Embase, Web of Science, ProQuest, EBSCO, PubMed, CINAHL, Cochrane Library, A+ Education and Scopus. The database searches produced *n* = 6024, which were reduced to *n* = 4959 after irrelevant and duplicate records were removed. See Figure 1 for the PRISMA diagram for the study identification and selection process.

### 2.1. Study Selection

The titles and abstracts of *n* = 4959 articles were reviewed against the inclusion and exclusion criteria. The Participants, Interventions, Comparators, Outcomes and Study Design (PICOs) tool was used to review studies (Methley et al., 2014). ‘Participants’ included secondary school teachers. ‘Interventions’ included targeted mental health literacy programs about depression, anxiety, psychosis or suicide risk. ‘Comparators’ included no intervention, other intervention, wait-list control. ‘Outcomes’ included measures evaluating teachers’ knowledge of depression, anxiety, psychosis or suicide risk. ‘Study design’ included randomized or quasi-randomized controlled trials, pre- and post-test designs, mixed methods or qualitative study designs.

Articles that met the inclusion criteria were those published in English, published in a peer-reviewed journal, occurring between the years 2000 and 2024, and evaluating teacher literacy/knowledge of signs and symptoms of depression, anxiety, psychosis or suicide risk. A teacher was defined as a professional educator whose main role was teaching adolescents aged 11–18 years at a secondary school (Year 7–12). Studies conducted in countries classified as developing economies according to the OECD were excluded because mental healthcare and education systems may be considerably different in those countries and access to basic health and education services can be limited (Alemayehu et al., 2018; OECD, 2012). Exclusion criteria are stated in Figure 1. Reviews against the exclusion criteria (see Figure 1) led to the identification of *n* = 156 articles. The full text of the articles was then reviewed, resulting in the inclusion of 18 articles.

### 2.2. Appraisal of Evidence

Two independent reviews (SB, CM) appraised the evidence using the McMaster Critical Review Form for Quantitative Studies (Law and MacDermid, 1998), the McMaster Critical Review Form for Qualitative Studies (Letts et al., 2007) and the Mixed Methods Appraisal Tool (MMAT) (Hong et al., 2018; Law & MacDermid, 1998). These appraisal tools were used because they are comprehensive in assessing methodological quality of evidence and have good inter-rater reliability (Law and MacDermid, 1998). A percentage score between the two reviewers was calculated for reliability (Viera and Garrett, 2005). Disagreements were resolved through discussion.

Both reviewers had experience with inter-rater reliability through evaluating studies in previous reviews. CM was the more experienced reviewer (holding a professorial qualification) and provided guidance as required. Pilot testing was conducted to ensure a common understanding of the rating criteria for the instruments used. Inter-reviewer reliability was ensured through discrepancy resolution when required, which was addressed via consensus, whereby the reviewers discussed the conflicting ratings to reach a shared understanding and agreement. Any disagreement relating to the inclusion of articles by reviewers was resolved by the reviewers reaching consensus after discussing whether the article met the inclusion criteria or should be excluded.

Study quality assessment was used to establish a minimum quality threshold for the selection of studies for the current review, investigate quality differences in study results, weigh study results proportionate to study quality, direct interpretation of findings, help assess the strength of inferences and direct recommendations for future research

The McMaster Critical Review Form for Quantitative Studies considers 16 items of methodological quality, including the aims of the study, design, intervention, outcome measures, statistical analysis and significance and clinical implications (Law and MacDermid, 1998). The McMaster Critical Review Form for Qualitative Studies considers 13 items of methodological quality, including procedural rigor, data analyses, theoretical connections, results and conclusions (Letts et al., 2007).

In this review, guidelines for evidence appraisal were created for both of these tools so that reviewers could consistently evaluate methodological quality (Table 1 and Table 2) based on McMaster appraisal tools (Law and MacDermid, 1998; Law et al., 1998; Letts et al., 2007). Studies were assessed against the predefined criteria of the guidelines, receiving a score of ‘1’ for each criterion met and ‘0’ if not met. Individual scores were summed to yield a total methodological quality score, with a maximum of 16 for quantitative studies and 13 for qualitative studies; higher scores indicate greater quality. The resulting total scores were then categorized into four groups for interpretation: Low quality (less than 40% correct), Fair (40.1% to 74.9% correct), Moderate (75.0% to 79.99% correct) and High (80% or above correct). The results of the appraisals are shown in Table 3 and Table 4.

### 2.3. Charting the Data and Results

The summary details of selected articles are presented in Table S1 (see Supplementary Materials), including first author, year of publication, country where study was conducted, urban or rural location, purpose, study design, data collection method, method, measures used, participant details (teachers, students, schools), mental health literacy program implemented, overall findings and study limitations.

## 3. Results

### 3.1. Methodological Quality

There was 88% agreement (14/16 articles) between reviewers about the item scores of the McMaster Critical Review Form for Quantitative Studies relating to methodological quality. Discussion between reviewers was required to reach consensus about whether there was enough information provided about depression and anxiety psychoeducational content included in the training in the studies conducted by O’Dea et al. (2023) and Parker et al. (2021) (Table 2).

The quantitative studies reviewed were found to have a varying methodological quality, with scores on the McMaster Quantitative Review Form ranging from 8 to 15 points (out of a potential 16), indicating an overall quality range from “Fair” to “High” (as detailed in Table 2). The strengths identified across the studies were related to the clearly defined purpose of the research (Item 1), strong justification for conducting the study (Item 2), the study design used (Item 3), and discussion of the clinical implications derived from the findings (Item 15). The majority of the studies presented results in terms of statistical significance (Item 11) and utilized appropriate statistical analyses to interpret the data (Item 12). All included studies failed to implement assessor blinding (Item 4). The majority of the studies did not meet the criteria for recruiting an adequate sample size (Item 5) or for sufficiently documenting the reliability of their outcome measures (Item 9). No studies were rated as ‘Low’ quality, five studies received a ‘Fair’ rating, five studies received a ‘Moderate’ rating and six studies received a ‘High’ methodological quality score.

There was a 100% inter-rater reliability for item scores of the McMaster Critical Review Form for Qualitative Studies. The methodological quality of the one included qualitative study (Shilubane et al., 2015) scored 7, representing “Fair” methodological quality (Table 4). According to the McMaster Qualitative Review Form, strengths in the methodological quality of the study were related to the provision of a clear purpose (Item 1), justification of the study (Item 2), study design (Item 3), the sample description (Item 5) and the documentation of ethics approval and consent (Item 7). This study did not, however, provide a theoretical perspective (Item 4) or outline clear data collection methods (Item 8) or procedural rigor (Item 9) (Table 4).

There was also 100% inter-rater reliability regarding the item scores of the Mixed Methods Appraisal Tool (MMAT) (Table 5). Strengths in the methodological quality of the one included mixed-method study (Exner-Cortens et al., 2022) included a clear research question and that the data collected addressed the research question. The qualitative component scored 3 out of 5 for quality criteria, and the quantitative description also scored 3 out of 5 for quality criteria. The mixed-method component scored 3 out of 5, as a rationale for a mixed-method design was not clearly outlined and the integration of qualitative and quantitative research was not clearly addressed.

The Mixed Methods Appraisal Tool (MMAT) (Hong et al., 2018) was utilized to appraise the mixed-method study included in this review. The authors of this tool do not recommend calculating an overall quality score for studies, but instead recommend providing ratings for each criterion, as seen in Table 5.

Sampling bias was frequently acknowledged in studies as a limitation because the characteristics and behaviors of the schools and teachers who volunteered for the studies may be different from those of the schools that chose not to participate. Ten studies included both teachers and other school staff as participants; therefore, the literacy results were not specific to secondary school teachers. The varied professions of participants within schools contribute to methodological heterogeneity.

All included studies using self-report measures acknowledged that social desirability bias may exist. Thirteen studies designed their own survey instrument, contributing to methodological heterogeneity. Only one study reported whether there were adverse events resulting from training (Jorm et al., 2010). Methodological heterogeneity in the use of standardized measures made it difficult to generalize the findings.

Vignettes were utilized in three studies as a method of assessing teachers’ ability to identify and help students with depression, anxiety and psychosis (Arslan & Karabey, 2023; Jorm et al., 2010; Vieira et al., 2014). The researchers acknowledged that participants’ responses to vignettes may differ from how they would respond in real life, and data collected may have been impacted by social desirability bias. Teachers’ intended behavior may differ from their actual behavior when confronted with a potentially mentally ill student or an individual at suicide risk.

Participants lost to follow-up and therefore unable to complete post-training measures can decrease the external validity of a study. In this review, six studies reported high attrition rates, which impacts the reliability of results (Bockhoff et al., 2022; Exner-Cortens et al., 2022; Miller et al., 2019; O’Dea et al., 2023; Parker et al., 2021; Wyman et al., 2008).

Five non-blinded cluster RCTs, randomized by school, were included in this review, with teachers being aware of the intervention given, which could result in bias and lead to deviation away from the true effect of a training intervention (Higgins et al., 2024). Awareness of the training intervention can result in performance bias and, therefore, impact the fidelity of the trial.

Different teacher training strategies and methods were implemented across the included studies, limiting the comparisons between studies and resulting in between-study heterogeneity. When comparing different studies, differences existed in study populations, methodologies, measures used and training interventions implemented. This heterogeneity reduces the ability to draw meaningful conclusions and generalize findings; therefore, combined results are not reliable and studies are not comparable.

### 3.2. Study Characteristics

#### 3.2.1. Country of Study

The eighteen studies were conducted in nine different countries, with four conducted in Australia (Jorm et al., 2010; O’Dea et al., 2023; Parker et al., 2021; Robinson et al., 2016), three conducted in Canada (Exner-Cortens et al., 2022; Wei and Kutcher, 2014; Wei et al., 2021), five conducted in the USA (Johnson & Parsons, 2012; Lamis et al., 2017; Miller et al., 2019; Reis & Cornell, 2018; Wyman et al., 2008), and one conducted in the UK (Moor et al., 2007). One study was conducted in Turkey (Arslan & Karabey, 2023), Germany (Bockhoff et al., 2022), South Africa (Shilubane et al., 2015), Brazil (Vieira et al., 2014), and Japan (Yamaguchi et al., 2023).

#### 3.2.2. Study Designs

Sixteen quantitative studies, one qualitative study (Shilubane et al., 2015) and one mixed-method study (Exner-Cortens et al., 2022) met the inclusion criteria. Of the quantitative studies, six implemented survey methodology (Arslan & Karabey, 2023; Johnson & Parsons, 2012; Lamis et al., 2017; Vieira et al., 2014; Wei et al., 2021; Yamaguchi et al., 2023), five studies were RCTs (Jorm et al., 2010; Miller et al., 2019; Moor et al., 2007; O’Dea et al., 2023; Wyman et al., 2008), two studies used pre- and post-test design (Robinson et al., 2016; Wei & Kutcher, 2014) and one study employed a quasi-experimental design (Reis & Cornell, 2018). One study had a two-factor experimental design (Bockhoff et al., 2022) and one utilized an uncontrolled single-arm pilot study (Parker et al., 2021).

Eight included studies evaluated the effectiveness of teacher mental health literacy training, collecting data before and immediately after the training (Miller et al., 2019; Moor et al., 2007; Reis & Cornell, 2018; Robinson et al., 2016; Vieira et al., 2014; Wei et al., 2021; Wei and Kutcher, 2014). Four studies evaluated teachers’ knowledge 3 to 6 months after training (Bockhoff et al., 2022; Johnson & Parsons, 2012; Jorm et al., 2010; O’Dea et al., 2023; Parker et al., 2021), while one study conducted an evaluation one year post-training (Wyman et al., 2008).

#### 3.2.3. Participants

The number of secondary school teachers participating in the included studies ranged from 25 to 857. In addition to secondary school teachers, one study included primary school teachers (Exner-Cortens et al., 2022), one included school social workers (Bockhoff et al., 2022), and seven included mental health support staff (Lamis et al., 2017; O’Dea et al., 2023; Reis & Cornell, 2018; Robinson et al., 2016; Wei et al., 2021; Wei and Kutcher, 2014; Wyman et al., 2008). Seven studies included students as participants, with sample sizes ranging from 52 to 6679 (Arslan & Karabey, 2023; Bockhoff et al., 2022; Jorm et al., 2010; Miller et al., 2019; Moor et al., 2007; Vieira et al., 2014; Wyman et al., 2008). Fourteen studies included the number of schools participating, ranging from 1 to 140 (Bockhoff et al., 2022; Johnson & Parsons, 2012; Jorm et al., 2010; Miller et al., 2019; Moor et al., 2007; O’Dea et al., 2023; Parker et al., 2021; Reis & Cornell, 2018; Robinson et al., 2016; Shilubane et al., 2015; Vieira et al., 2014; Wei and Kutcher, 2014; Wyman et al., 2008; Yamaguchi et al., 2023).

#### 3.2.4. Training

Two hours or less of training were implemented and evaluated in four studies (Exner-Cortens et al., 2022; Johnson & Parsons, 2012; Moor et al., 2007; Wyman et al., 2008). Between 2 to 8 hours of training were implemented in six studies (Miller et al., 2019; Reis & Cornell, 2018; Vieira et al., 2014; Wei et al., 2021; Wei and Kutcher, 2014; Parker et al., 2021), and three studies implemented and evaluated between 9 and 14 h of training (Bockhoff et al., 2022; Jorm et al., 2010; Robinson et al., 2016). Training was delivered by external professionals, who included researchers, in some studies (see Table S1 in the Supplementary Materials). Teachers did not receive any mental health training in three studies because only current mental health literacy was assessed (Arslan & Karabey, 2023; Shilubane et al., 2015; Yamaguchi et al., 2023).

#### 3.2.5. Studies Measuring Knowledge of Depression, Anxiety, Psychosis or Suicide Risk

Nine studies measured teachers’ knowledge of depression (Arslan & Karabey, 2023; Jorm et al., 2010; Miller et al., 2019; Moor et al., 2007; O’Dea et al., 2023; Parker et al., 2021; Vieira et al., 2014; Wei et al., 2021; Wei and Kutcher, 2014). Five studies measured teachers’ knowledge of an anxiety disorder (Arslan & Karabey, 2023; Jorm et al., 2010; O’Dea et al., 2023; Wei et al., 2021; Wei and Kutcher, 2014), while five studies measured knowledge of psychosis (Arslan & Karabey, 2023; Jorm et al., 2010; Vieira et al., 2014; Wei et al., 2021; Wei and Kutcher, 2014) and nine studies measured teachers’ knowledge of suicide risk (Bockhoff et al., 2022; Exner-Cortens et al., 2022; Johnson & Parsons, 2012; Lamis et al., 2017; Reis & Cornell, 2018; Robinson et al., 2016; Shilubane et al., 2015; Wyman et al., 2008; Yamaguchi et al., 2023).

#### 3.2.6. Urban or Rural Schools

Four studies were conducted in urban schools (Arslan & Karabey, 2023; Bockhoff et al., 2022; Robinson et al., 2016; Vieira et al., 2014), while five studies were conducted in both urban and rural schools (Exner-Cortens et al. 2022; Jorm et al., 2010; O’Dea et al., 2023; Parker et al., 2021; Reis & Cornell, 2018). The one qualitative study was conducted in a rural school (Shilubane et al., 2015), while eight studies did not report whether they were conducted in rural or urban schools.

### 3.3. Teachers’ Mental Health Literacy

Mental health literacy training was delivered to teachers and evaluated in fifteen studies. Teachers’ existing knowledge was evaluated in three studies, with no training provided (Arslan & Karabey, 2023; Shilubane et al., 2015; Yamaguchi et al., 2023).

#### 3.3.1. Depression Literacy

Nine studies measured teachers’ knowledge of depression (Arslan & Karabey, 2023; Jorm et al., 2010; Miller et al., 2019; Moor et al., 2007; O’Dea et al., 2023; Parker et al., 2021; Vieira et al., 2014; Wei et al., 2021; Wei and Kutcher, 2014) (Table 6). The training packages that taught the signs and symptoms of depression were Mental Health First Aid (Jorm et al., 2010), the Adolescent Depression Awareness Program (ADAP) (Miller et al., 2019), the Building Educators Skills in Adolescent Mental Health (BEAM) (O’Dea et al., 2023; Parker et al., 2021) and the ‘Go-To’ Educator Training (Wei et al., 2021; Wei and Kutcher, 2014). Two studies implemented their own researcher-designed training package on adolescent depression (Moor et al., 2007; Vieira et al., 2014). Arslan and Karabey (2023) evaluated teachers’ existing knowledge of adolescent depression without implementing a training package. The instruments used to measure knowledge of depression included established instruments, such as the Mental Health Literacy Questionnaire (Jorm et al., 1997), the Adolescent Depression Knowledge Questionnaire (Hart et al., 2014; Miller et al., 2019) and the Mental Health Knowledge and Stigma Survey (Wei and Kutcher, 2014). Some studies used questionnaires about vignettes depicting depression (Arslan & Karabey, 2023; Moor et al., 2007; Vieira et al., 2014) and researcher-developed scales (Jorm et al., 2010; O’Dea et al., 2023; Parker et al., 2021).

Arslan and Karabey (2023) found that 45.5% of teachers correctly identified student depression in the vignette and 90% believed the student needed a referral to a psychologist. Jorm at al. (2010) found that recognition of depression in a student vignette was high at pre-test and was not affected by the training. The ADAP training program study conducted by Miller et al. (2019) found that post-training, teachers scored an average of 93.5% on the Adolescent Depression Knowledge Questionnaire. This study also reported that teachers’ depression literacy was significantly associated with students’ depression literacy (*p* = 0.035), indicating that teachers’ knowledge about depression could influence students’ knowledge. Miller et al. (2019) evaluated the effectiveness of the psychoeducational intervention designed to help teachers recognize the symptoms of clinical depression in their students. Students were screened for depression, and the evaluation of the program showed that teachers’ ability to recognize depressed students did not improve. Parker et al. (2021) found no significant changes in knowledge or attitudes after the BEAM training and 3-month follow-up; however, significant increases in confidence and helping behaviors occurred. Both Wei et al. (2021) and Wei and Kutcher (2014) did not specifically provide results about teachers’ knowledge of depression post-training; however, both studies reported that teachers’ overall knowledge improved significantly post-training (*p* < 0.001).

#### 3.3.2. Anxiety Literacy

Five studies measured teachers’ knowledge of an anxiety disorder (Arslan & Karabey, 2023; Jorm et al., 2010; O’Dea et al., 2023; Wei et al., 2021; Wei and Kutcher, 2014) (Table 7). Psychoeducational training packages focusing on signs and symptoms of anxiety included Mental Health First Aid (Jorm et al., 2010), Building Educators Skills in Adolescent Mental Health (BEAM) (O’Dea et al., 2023) and the ‘Go-To’ Educator Training (Wei et al., 2021; Wei and Kutcher, 2014). Arslan and Karabey (2023) measured teachers’ existing knowledge about social phobia by way of a vignette without any training provided. Instruments used to measure teachers’ knowledge of anxiety included the Mental Health Literacy Questionnaire (Jorm et al., 1997), Knowledge of Mental Health Problems Questionnaire (Jorm et al., 2010), the Confidence in Recognizing and Responding to Students’ Mental Health Needs (O’Dea et al., 2023) questionnaires and the Mental Health Knowledge and Stigma Survey (Wei et al., 2021; Wei and Kutcher, 2014).

Arslan and Karabey (2023) found that 27.9% of the teachers correctly identified the vignette presenting a student with a social phobia, and 90% believed they needed a referral to a psychologist. Though anxiety knowledge scores were not specifically mentioned in the results, Jorm at al. (2010) reported that the training increased teachers’ knowledge significantly, and this knowledge was sustained 6 months after training. O’Dea et al. (2023) found that teachers who received the BEAM training program reported significantly higher levels of confidence, perceived mental health knowledge and awareness at post-intervention and 3-month follow-up relative to the control group. No significant effects were found for teachers’ helping behaviors. Both Wei et al. (2021) and Wei and Kutcher (2014) did not specifically provide results about teachers’ knowledge of anxiety disorders post-training; however, both studies reported teachers’ overall knowledge improved significantly post-training (*p* < 0.001).

#### 3.3.3. Early Psychosis Literacy

Five studies measured teachers’ knowledge of psychosis (Arslan & Karabey, 2023; Jorm et al., 2010; Vieira et al., 2014; Wei et al., 2021; Wei and Kutcher, 2014) (Table 8). Arslan and Karabey (2023) evaluated teachers’ existing knowledge of psychosis by providing a vignette and an associated questionnaire. In the study by Jorm et al. (2010), Mental Health First Aid training taught teachers how to identify and give initial help to students who were experiencing a psychosis. This knowledge was evaluated using the Knowledge of Mental Health Problems Questionnaire (Jorm et al., 2010). Vieira et al. (2014) provided a psychoeducational training package developed by the researchers about psychosis. Teachers’ knowledge of psychosis was evaluated through a questionnaire using vignettes. Wei et al. (2021) and Wei and Kutcher (2014) implemented ‘Go-To’ Educator Training, which included psychoeducational information about psychosis. Both these studies measured teachers’ knowledge following training using the Mental Health Knowledge and Stigma Survey (Wei and Kutcher, 2014).

Arslan and Karabey (2023) found that 47.9% of their sample were able to correctly identify a student with psychosis, and 90.9% identified a psychiatrist as the person to refer the student to. Though psychosis knowledge scores were not specifically mentioned in the results, Jorm et al. (2010) reported that the training increased teachers’ overall knowledge significantly. Vieira et al. (2014) found that prior to training, 76.7% of teachers identified psychosis correctly. The researchers found that most teachers could already identify students experiencing psychosis. Wei et al. (2021) and Wei and Kutcher (2014) did not report on teachers’ knowledge of psychosis specifically in their results.

#### 3.3.4. Suicide Risk Literacy

Nine studies measured teachers’ knowledge of suicide risk (Bockhoff et al., 2022; Exner-Cortens et al., 2022; Johnson & Parsons, 2012; Lamis et al., 2017; Reis & Cornell, 2018; Robinson et al., 2016; Shilubane et al., 2015; Wyman et al., 2008; Yamaguchi et al., 2023) (Table 9). Training packages implemented with teachers included the Question, Persuade, Refer training (QPR, Quinnett, 1995) (Bockhoff et al., 2022; Exner-Cortens et al., 2022; Johnson & Parsons, 2012; Reis & Cornell, 2018; Wyman et al., 2008), STORM (Appleby et al., 2000) (Robinson et al., 2016) and Making Educators Partners in Suicide Prevention (MEP) (Lamis et al., 2017). Two studies did not implement training; instead, they evaluated current teacher knowledge (Shilubane et al., 2015; Yamaguchi et al., 2023). Methods of evaluating knowledge varied across studies, with most studies developing their own instrument (Bockhoff et al., 2022; Exner-Cortens et al., 2022; Johnson & Parsons, 2012; Lamis et al., 2017; Reis & Cornell, 2018; Robinson et al., 2016; Wyman et al., 2008; Yamaguchi et al., 2023). Bockhoff et al. (2022) also used vignettes to assess whether teachers could identify suicide risk, while Wyman et al. (2008) used the standardized Knowledge of QPR Instrument (Quinnett, 1999) to measure teacher knowledge. Shilubane et al. (2015) implemented focus groups with teachers to measure knowledge of suicide risk; however, no training was provided.

All studies evaluating QPR training for suicide prevention reported significant results for improving teachers’ knowledge of suicide risk (Bockhoff et al., 2022; Exner-Cortens et al., 2022; Johnson & Parsons, 2012; Reis & Cornell, 2018). STORM training also resulted in increased teacher knowledge and confidence scores from pre- and post-training measurements (Robinson et al., 2016). Results indicated that Making Educators Partners in Suicide Prevention (MEP) training also demonstrated significant increases in teachers’ suicide knowledge, attitudes and self-efficacy (Lamis et al., 2017). Shilubane and colleagues (2015) identified themes related to teachers’ knowledge of warning signs for suicidal behaviors, the experience of a student death by suicide and the emotional consequences. Teachers were not well informed about the warning signs of suicide and expressed the need to be able to identify students at risk and refer them to services for help. Yamaguchi et al. (2023) found that suicide literacy in Japanese school teachers was limited. The average proportion of correct answers to the knowledge questions (10 items) was 55.2%.

The findings of the review highlighted strengths and weaknesses in teacher’ disorder-specific mental health literacy training and effectiveness. There was varying quality among the quantitative studies, with generally inadequate sample sizes, and most outcome measures not having established reliability. Fifteen studies measured mental health literacy training effectiveness, with some using established measurement instruments, and others using researcher-developed scales. Teacher training related to anxiety increased knowledge, which was reported to be sustained over time in some studies. While a range of training programs were used to teach teachers the signs and symptoms of depression, most training improved teachers’ mental health literacy. Teacher identification of psychosis increased after training, and most were able to identify students experiencing psychosis and the need for referral to a psychiatrist. Training programs such as QPR, STORM and the MEP increased teacher knowledge of suicide risk, and STORM increased teacher confidence.

## 4. Discussion

When healthcare systems lack sufficient capacity to provide rapid assessment and follow-up care for mentally unwell adolescents, schools are often required to function as de facto frontline sites for recognition and triage. Within these systemic conditions, the expectation is that schools play an increasingly large “system compensation” role in identifying, supporting and responding to student mental illness, imposing additional demands on secondary school teachers (Productivity Commission, 2020). There is, therefore, a recognized need to improve teacher training in student mental illness, to promote early intervention and to improve knowledge, attitudes, decision-making and helping behaviors towards those students in need (Kelly, 2011; Mei et al., 2020; McGorry et al., 2022; Productivity Commission, 2020).

The focus of this review aligns with Bandura’s Social Cognitive Theory (Bandura, 1977, 2006), specifically focusing on teachers’ self-efficacy, defined as the “teacher’s belief in their capability to organize and execute courses of action required to successfully accomplish a specific task” (Tschannen-Moran et al., 1998, p. 233). Research that has investigated the concept of teacher self-efficacy in supporting student mental illness has found that high levels of self-efficacy depend on training, clear role definition (Watson, 2024) and confidence in identifying students with mental illness (Heng & Chu, 2023). Studies have found that most teachers take student mental health issues very seriously but report that their previous training in the area has been inadequate (Frauenholtz et al., 2017; Moon et al., 2017). This review advances theory, specifically by identifying gaps in the literature and suggesting ways to improve teachers’ mental health literacy in order to improve teachers’ self-efficacy in supporting students with mental illness.

Depression and anxiety show high prevalence amongst adolescents, frequently occurring whilst they are in secondary school, and are a leading contributor to the burden of disease for this age group (AIHW, 2025; Lawrence et al., 2015; Robson et al., 2024; WHO, 2023). Early psychosis is not as prevalent as depression or anxiety in this age range; however, research has shown that a large percentage of those who experience it do not finish school (Bowman et al., 2014; Goulding et al., 2010; Waghorn et al., 2012). This suggests that early symptoms may exist whilst adolescents are in school. Increasing teachers’ literacy about early psychosis may promote early intervention and better academic outcomes. Given that suicide is the third leading cause of death for 15–19-year-olds across the world and the leading cause of death for this age range in Australia, suicide risk literacy is extremely important for secondary school teachers (AIHW, 2025; WHO, 2020). Teachers with high levels of disorder-specific mental health literacy may be able to recognize and refer students for early intervention (Miller et al., 2019). This review addressed the gap in the literature by focusing specifically on teachers’ disorder-specific literacy for student depression, anxiety, early psychosis and suicide risk. Nine studies in this review evaluated teachers’ knowledge of student depression, five evaluated knowledge of anxiety, five evaluated psychosis and nine focused on suicide risk.

There was variation between the studies in training content and modalities, length of training provided, evaluation measures used and training facilitation methods. There was a high risk of bias and methodological heterogeneity in the included studies, which supports findings from previous systematic reviews (Anderson et al., 2019; Ohrt et al., 2020; Prabhu et al., 2024; Yamaguchi et al., 2020). A small number of studies measured teachers’ literacy about anxiety and early psychosis.

### 4.1. Depression

While nine included studies measured teachers’ depression literacy, only two studies specifically focused training on adolescent depression (Miller et al., 2019; Moor et al., 2007). Moor and colleagues implemented a 2 h depression training package for teachers and then evaluated their ability to identify students experiencing it. They found the training did not improve their ability to do so. Previous research has found that teacher professional development needs to be sustained over time to be effective (Sims & Fletcher-Wood, 2021). It takes time for teachers to learn, and single-day training sessions are often ineffective (Sims & Fletcher-Wood, 2021). The training package in this study may have been too short, and if it was provided over time, it may have improved results. Miller et al. (2019) implemented a 6 h depression-specific training program, which resulted in teachers scoring an average of 93.5% of questions correctly on the Adolescent Depression Knowledge Questionnaire (Hart et al., 2014). This study had a small sample size and warrants future research using a bigger sample. Additional research is needed to investigate whether the training results in increased helping behaviors by teachers towards depressed students and whether knowledge improves decision-making, confidence and navigation of the referral pathways required.

### 4.2. Anxiety

Of the five studies that measured teachers’ literacy of student anxiety, only one study, which measured knowledge about social phobia by way of vignette, provided a specific result, with 27.9% of teachers answering correctly (Arslan & Karabey, 2023). Though Jorm et al. (2010), O’Dea et al. (2023), Wei et al. (2021) and Wei and Kutcher (2014) included anxiety literacy in their training packages, they did not report specific results about teachers’ anxiety literacy. Further research is required to develop an effective training package for teachers on adolescent anxiety and evaluate it using a specific measure focusing on knowledge of anxiety disorders (Kessler et al., 2007; Lawrence et al., 2015; AIHW, 2024).

Researchers have found that professional development training should include opportunities to practice and apply what has been learned so teachers know how to use the new knowledge in real classroom situations (Cordingley, 2015; Dunst et al., 2015). Sims and Fletcher-Wood (2021) proposed that professional development should upskill teachers so that they gain a deeper understanding of how student mental ill health can impact teaching and learning, and provide strategies to address it. This approach is in contrast to training via lectures, where teachers receive new information but do not learn how to apply it (Sims & Fletcher-Wood, 2021). These characteristics of effective professional development training programs for teachers could be implemented in future training packages on anxiety.

### 4.3. Early Psychosis 

None of the included studies focused on early psychosis literacy alone. Studies conducted by Arslan and Karabey (2023) and Vieira et al. (2014) used vignettes to evaluate teachers’ ability to recognize students experiencing psychosis. Arslan and Karabey (2023) found that 47.9% of their sample were able to correctly identify a student with psychosis, while Vieira et al. (2014) found that prior to training, 76.7% of teachers identified psychosis correctly.

No other included studies provided results specific to psychosis literacy, even though psychosis was part of their training package (Jorm et al., 2010; Wei et al., 2021; Wei and Kutcher, 2014). Further research is required to investigate the effectiveness of a targeted early psychosis training package on teachers’ knowledge, confidence in dealing with a student who may be experiencing psychosis, and effective navigation of referral pathways. A specific measure evaluating the effectiveness of early psychosis training for teachers that can be used consistently across studies is also required. Receiving training from outside experts could increase the effectiveness of teacher training (Cordingley, 2015). This could be considered when planning teacher training on early psychosis.

### 4.4. Suicide Risk

All included studies found that training significantly improved teachers’ knowledge of suicide risk. The next step in this area is to provide training that improves teachers’ confidence about what to do once a student is identified, who to refer to, and what steps to take to ensure a student’s safety. Robinson and colleagues, in their study investigating the effectiveness of STORM training, used filmed roleplays as one of their outcome measures pre- and post-training, with an actor playing a student who had engaged in self-harm. Teachers were required to assess their suicide risk, and it was found that teachers asked more specific suicide-risk assessment questions after training. This strategy aligns with evidence from professional development research that suggests training should include opportunities to practice and apply what has been learned (Cordingley, 2015; Dunst et al., 2015; Sims et al., 2023). Teachers’ suicide risk literacy should include guidance on what specifically to do to ensure the suicidal student is safe and referred for help. This requires further development and investigation.

Research has found that training is more effective when it involves a subject that is meaningful to the teacher (Cordingley, 2015). Providing teachers with training on depression, anxiety, early psychosis and suicide risk rather than with training on general mental health literacy, and evaluating their knowledge of these specific subjects, which this review has identified as the preferred approach in previous studies, is recommended for future research. Knowledge gaps can be identified and addressed. Training to increase literacy on depression, anxiety, early psychosis and suicide risk might be ineffective if it fails to change teachers’ understanding of how to apply it to teaching and learning.

There is limited focus in existing evidence on how knowledge improves the helping behavior of teachers towards students in need. This finding is in agreement with previous reviews (Anderson et al., 2019; Ohrt et al., 2020; Yamaguchi et al., 2020). Future research should include outcome measures to evaluate whether improved teachers’ literacy about depression, anxiety, early psychosis and suicide risk leads to better mental health outcomes for young people, such as students accessing early intervention. Longitudinal studies are required to investigate whether the effects of the literacy training sessions are sustained, with a particular focus on the required frequency of training to maintain knowledge. Co-design studies with teachers could further inform the research agenda in this area.

The integration of disorder-specific mental health literacy in student teachers’ training curricula and as part of faculty development programs should be considered. Evaluation of disorder-specific literacy training provided in pre-service and undergraduate teacher training programs is needed to ensure graduate teachers are informed and skilled in strategies to manage student mental ill health in the classroom. This may assist them in coping with the requirements of the real-world classroom. The literacy of teachers in developing countries should also be further investigated.

Future studies could consider strategies for reducing risk of bias, such as selecting participants, controlling for confounding variables, concealing allocation to the intervention or control group, using an active control group to address limitations and measuring disorder-specific knowledge. Many studies included in this review had a risk of bias because they did not randomly recruit participants or provide adequate recruitment details. Most studies did not control for possible confounding variables, and few validated questionnaires were used, making it difficult to assess the generalizability of their results. Measures of program fidelity were not utilized in the studies; therefore, it is unclear if the programs were administered in accordance with the protocol and consistently.

### 4.5. Limitations

This scoping review aimed to identify the types of evidence available, determine gaps in the literature and better understand how research has been conducted relating to teachers’ depression, anxiety, psychosis and suicide risk literacy. This scoping review did not combine statistical data from studies to develop synthesized results, which is a limitation, and focused only on identifying the breadth of information on the topic rather than depth (Campbell et al., 2023).

A significant limitation in this review is language bias, as only English-language peer-reviewed articles were included. This may have skewed the results and affected generalizability, as crucial data or findings may have appeared in non-English studies. This review only included peer-reviewed journal articles, excluding editorials/theses/grey literature, potentially resulting in selection bias by missing relevant studies and reducing generalizability. This review also only included predominantly Western contexts. Restricting the review to Western contexts is a significant limitation, introducing selection bias, affecting generalizability and presenting a skewed understanding by overlooking non-Western evidence. Of the included studies, 9 were published more than 10 years ago, and 5 studies were more than 5 years old. Including studies published prior to the COVID-19 pandemic may have limited generalizability because the workforce and school systems have changed since those studies were conducted. Teachers’ roles and experiences may be different now.

This review was descriptive in nature, and many studies did not meet the final inclusion criteria of the review, with only 18 of the 4959 studies meeting all the necessary requirements for inclusion.

Only 1 study included in this review was conducted in a rural school, while 6 studies included both rural and urban schools but did not report specific knowledge data and results for rural participants. The disorder-specific knowledge of rural secondary school teachers is largely unknown.

## 5. Conclusions

No previous review, to our knowledge, has focused on mental disorder–specific literacy of secondary school teachers. This review addressed the gap by describing and reporting on the depression, anxiety, early psychosis and suicide risk literacy of secondary school teachers. This review found that there were a limited number of studies focusing on teachers’ disorder-specific literacy. The training provided in the included studies was mostly combined into a general mental health literacy package, and disorder-specific knowledge was rarely measured; as a result, teachers’ knowledge gaps are largely unknown. Suicide risk training results in improved teacher knowledge in the short term, but there is no evidence that this knowledge is sustained over time or translated into helping behaviors. Most included studies implemented less than 8 h of training, and teachers may need longer and more frequent training. Increasing their literacy about depression, anxiety, early psychosis and suicide risk might be ineffective if it fails to change their understanding of how to apply it to teaching and learning. Training should include what steps are needed once teachers identify a student in need or at risk. These recommendations should be considered in future studies investigating the mental health literacy of teachers. Considering the high prevalence of adolescent depression and anxiety, the profound functional consequences of untreated early psychosis and suicide being the number one cause of death in Australia for 15–19-year-olds, the literacy of teachers about these disorders should be a high priority. Teachers’ “system compensation” role requires not only improvements in knowledge but also competencies in decision-making, confidence and effectively navigating referral pathways. These skills may support teachers in dealing with the real-world demands of adolescent mental illness in secondary school.

## Figures and Tables

**Table 1:** Methodological quality of included studies—McMaster Critical Review Form for Quantitative Studies (Law and MacDermid, 1998).

Item	Essential Criteria
1. Purpose	Do the authors clearly state the aim of the study, which is to investigate the mental health literacy of secondary school teachers about adolescent depression, anxiety, early psychosis or suicide risk?
2. Literature review	Do the authors justify the need for the study, namely the need to undertake further research into the mental health literacy of secondary school teachers about adolescent depression, anxiety, early psychosis or suicide risk?
3. Study design	Have the authors described the study design to address the study aims, that is, to evaluate the mental health literacy of teachers?
4. Blinding	Have the authors used assessor blinding to minimize bias?
5. Sample description	Do the authors describe the sample in terms of age, gender and number of participants?
6. Sample set	Have the authors justified their sample size through a power calculation or post hoc analysis and recruited sufficient numbers?
7. Ethics and consent	Have the authors documented ethical approval for the research and gained informed consent from participants?
8. Validity of outcomes	Did the authors use valid outcome measures to measure the mental health literacy of secondary school teachers specifically about adolescent depression, anxiety, early psychosis or suicide risk?
9. Reliability of outcomes	Did the authors use reliable outcome measures to measure the mental health literacy of secondary school teachers specifically about adolescent depression, anxiety, early psychosis or suicide risk?
10. Intervention description	Did the authors provide sufficient information to enable reproduction of the intervention?
11. Statistical significance	Did the authors report on at least one outcome measure in line with the study aim and in terms of statistical significance?
12. Statistical analysis	Did the authors use appropriate statistical analyses in evaluating results according to their aim?
13. Clinical importance	Did the authors reflect on the clinical importance of the results for secondary school teachers?
14. Conclusions	Did the authors provide appropriate conclusions considering the study method and result?
15. Clinical implications	Did the authors discuss the implications of the results in terms of secondary school teachers’ mental health literacy about adolescent depression, anxiety, early psychosis or suicide risk?
16. Study limitations	Did the authors identify limitations of the study methodology and results?

**Table 2:** Modified guidelines for the use of the McMasters Critical Form for Qualitative Studies (Letts et al., 2007).

Item	Essential Criteria
1. Purpose	Do the authors clearly state the aim of the study and/or research question, which is to investigate the mental health literacy of secondary school teachers about adolescent depression, anxiety, early psychosis or suicide risk?
2. Literature review	Do the authors justify the need for the study? Was it clear and compelling—the need to undertake further research into the mental health literacy of secondary school teachers about adolescent depression, anxiety, early psychosis or suicide risk? Was relevant background literature reviewed?
3. Study design	Was the study design appropriate for the research question, that is, to investigate the mental health literacy of secondary school teachers about adolescent depression, anxiety, early psychosis or suicide risk?
4. Theoretical perspective	Was a theoretical perspective described? Are the methods congruent with the philosophical underpinnings and purpose?
5. Sample description	Have the authors described the sampling method used? Was the process of purposeful section described? Was sampling performed until redundancy in data was reached?
6. Sample set	Have the authors described the sample in adequate detail, in terms of demographic data and number of participants?
7. Ethics and consent	Have the authors documented ethical approval for the research and gained informed consent from participants?
8. Data collection	Were clear and complete descriptions of data collection provided? Were the role of researcher and the relationship with participants described? Were the assumptions and biases of researchers identified?
9. Procedural rigor	Do the authors provide adequate information about data collection procedures, e.g., gaining access to the site, field notes and training data gatherers?
10. Data analyses	Were the data analysis methods inductive? Were the findings consistent and reflective of the data? Was a decision trail developed and outlined? Was the process of analyzing the data described?
11. Theoretical connections	Were the concepts under study clarified and refined and relationships made clear? Did a meaningful picture of the phenomenon under study emerge?
12. Overall rigor	Was there evidence of the four components of trustworthiness—credibility, transferability, dependability and confirmability?
13. Conclusions and implications	Were the conclusions appropriate given the study findings? Were the clinical implications outlined? Were the limitations of the study discussed?

**Table 3:** Results of McMasters Critical Review Form for Quantitative Studies (Law and MacDermid, 1998).

Study	Individual Item																Total/16	Quality Descriptor
	1	2	3	4	5	6	7	8	9	10	11	12	13	14	15	16		
(Arslan and Karabey, 2023)	1	1	1	0	1	0	0	1	1	0	0	1	1	0	1	1	10	Fair
(Bockhoff et al., 2022)	1	1	1	0	0	0	0	1	1	0	1	1	1	1	1	1	11	Fair
(Johnson & Parsons, 2012)	1	1	1	0	0	0	0	1	0	1	0	0	1	1	1	0	8	Fair
(Jorm et al., 2010)	1	1	1	0	1	1	1	1	0	1	1	1	1	1	1	1	14	High
(Lamis et al., 2017)	1	1	1	0	1	1	0	1	1	1	1	1	1	0	1	1	13	High
(Miller et al., 2019)	1	1	1	0	0	0	1	1	1	1	1	1	1	1	1	1	13	High
(Moor et al., 2007)	1	1	1	0	0	0	1	1	1	1	1	1	1	0	1	1	12	Moderate
(O’Dea et al., 2023)	1	1	1	0	1	1	1	1	1	1	1	1	1	1	1	1	15	High
(Parker et al., 2021)	1	1	1	0	1	0	1	1	1	1	1	1	0	1	1	1	13	High
(Reis & Cornell, 2018)	1	1	1	0	0	0	0	1	0	1	1	1	1	1	1	1	11	Fair
(Robinson et al., 2016)	1	1	1	0	1	0	1	1	1	1	1	1	1	1	1	1	14	High
(Vieira et al., 2014)	1	1	1	0	1	0	1	1	0	0	1	1	1	1	1	1	12	Moderate
(Wei et al., 2021)	1	1	1	0	1	1	0	1	1	1	1	1	0	1	1	1	12	Moderate
(Wei and Kutcher, 2014)	1	1	1	0	0	0	1	1	0	1	1	1	1	0	1	1	11	Fair
(Wyman et al., 2008)	1	1	1	0	1	0	1	1	0	1	1	1	1	0	1	1	12	Moderate
(Yamaguchi et al., 2023)	1	1	0	0	1	0	1	1	1	0	1	1	1	1	1	1	12	Moderate

Scores “yes” = 1, “no” or “not addressed” = 0. Interpretation of final quality scores: Low quality: less than 40%; Fair: 40.1% to 74.9%; Moderate: 75.0% to 79.99%; High: 80% or above.

**Table 4:** Results of McMasters Critical Form for Qualitative Studies (Letts et al., 2007).

Study	Individual Item													Total/13	Quality Descriptor
	1	2	3	4	5	6	7	8	9	10	11	12	13		
(Shilubane et al., 2015)	1	1	1	0	1	0	1	0	0	0	0	1	1	7	Fair

Scores “yes” = 1, “no” or “not addressed” = 0. Interpretation of final quality scores: Low quality: less than 40%; Fair: 40.1% to 74.9%; Moderate: 75.0% to 79.99%; High: 80% or above.

**Table 5:** Results of the Mixed Methods Appraisal Tool (MMAT), version 2018.

Category of Study Methodological Quality Criteria	Yes	No	Can’t Tell
Study: (Exner-Cortens et al., 2022)			
S1. Are there clear research questions?	*		
S2. Do the collected data allow the research questions to be addressed?	*		
1. Qualitative Component of Study			
1.1. Is the qualitative approach appropriate to answer the research questions?	*		
1.2. Are the qualitative data collection methods adequate to address the research question?	*		
1.3. Are the findings adequately derived from the data?	*		
1.4. Is the interpretation of results sufficiently substantiated by data?			*
1.5. Is there coherence between qualitative data sources, collection, analysis and interpretation?			*
4. Quantitative Component of Study			
4.1 Is the sampling strategy relevant to address the question?	*		
4.2 Is the sample representative of the target population?	*		
4.3 Are the measurements appropriate?			*
4.4 Is the statistical analysis appropriate to answer the research question?	*		
5. Mixed Method Integration			
5.1 Is there an adequate rationale for using a mixed-method design to address the research question?		*	
5.2 Are the different components of the study effectively integrated to answer the research questions?	*		
5.3 Are the outputs of the integration of qualitative and quantitative components adequately interpreted?			*
5.4 Are divergencies and inconsistencies between quantitative and qualitative results adequately addressed?	*		
5.5 Do the different components of the study adhere to the quality criteria of each tradition of the methods involved?	*		

Scores * = yes.

**Table 6:** Depression studies—instruments used and training provided.

Study	Instrument Used to Measure Knowledge of Depression	Literacy Training Content
1. Arslan and Karabey (2023)	Mental Health Literacy Questionnaire (Jorm et al., 1997). Vignette presented of depression (Arslan & Karabey, 2023)	No training
2. Jorm et al. (2010)	Knowledge about mental health problems (Jorm et al., 2010) Recognition of depression in a vignette (Jorm et al., 2010) Stigma towards depressed students (Jorm et al., 2010) Beliefs about treatment of depression (Jorm et al., 2010) Intentions to help a depressed student (Jorm et al., 2010)	Mental Health First Aid
3. Miller et al. (2019)	The Adolescent Depression Knowledge Questionnaire (ADKQ) (Hart et al., 2014)	Adolescent Depression Awareness Program (ADAP)
4. Moor et al. (2007)	Teachers given class lists to indicate which students they believed were depressed (Moor et al., 2007)	Training package on adolescent depression developed by researchers
5. O’Dea et al. (2023)	Confidence in Recognizing and Responding to Students’ Mental Health Needs (O’Dea et al., 2023)	Building Educators Skills in Adolescent Mental Health Program (BEAM)
6. Parker et al. (2021)	Depression Stigma Scale (Parker et al., 2021)	Building Educators Skills in Adolescent Mental Health Program (BEAM)
7. Vieira et al. (2014)	Questionnaire with vignettes, one indicating high risk for depression (Vieira et al., 2014)	Training package on adolescent depression developed by researchers
8. Wei et al. (2021)	Mental Health Knowledge and Stigma Survey (measures knowledge about depression) (Wei and Kutcher, 2014)	‘Go-To’ Educator Training
9. Wei and Kutcher (2014)	Mental Health Knowledge and Stigma Survey (measures knowledge about depression) (Wei and Kutcher, 2014)	‘Go-To’ Educator Training

**Table 7:** Anxiety studies—instruments used and training provided.

Study	Instrument Used to Measure Knowledge of Anxiety	Literacy Training Content
1. Arslan and Karabey (2023)	Mental Health Literacy Questionnaire (Jorm et al., 1997) —vignette presented of an anxiety disorder (Arslan & Karabey, 2023)	No training
2. Jorm et al. (2010)	Knowledge about mental health problems (Jorm et al., 2010)	Mental Health First Aid
3. O’Dea et al. (2023)	Confidence in Recognizing and Responding to Students’ Mental Health Needs (O’Dea et al., 2023)	Adolescent Depression Awareness Program (ADAP)
4. Wei et al. (2021)	Mental Health Knowledge and Stigma Survey (measures knowledge about anxiety) (Wei and Kutcher, 2014)	‘Go-To’ Educator Training
5. Wei and Kutcher (2014)	Mental Health Knowledge and Stigma Survey (measures knowledge about anxiety) (Wei and Kutcher, 2014)	‘Go-To’ Educator Training

**Table 8:** Early psychosis studies—instruments used and training provided.

Study	Instrument Used to Measure Knowledge of Psychosis	Literacy Training Content
1. Arslan and Karabey (2023)	Mental Health Literacy Questionnaire (Jorm et al., 1997). Vignette of psychosis (Arslan & Karabey, 2023)	No training provided
2. Jorm et al. (2010)	Knowledge about mental health problems (Jorm et al., 2010)	Mental Health First Aid
3. Vieira et al. (2014)	Questionnaire with vignettes, one highlighting behavior indicating high risk for psychosis (Vieira et al., 2014)	Training package on early psychosis developed by researchers
4. Wei et al. (2021)	Mental Health Knowledge and Stigma Survey (measures knowledge about schizophrenia) (Wei and Kutcher, 2014)	‘Go-To’ Educator Training
5. Wei and Kutcher (2014)	Mental Health Knowledge and Stigma Survey (measures knowledge about schizophrenia) (Wei and Kutcher, 2014)	‘Go-To’ Educator Training

**Table 9:** Suicide risk studies—instruments used and training provided.

Study	Instrument Used to Measure Knowledge of Suicide Risk	Literacy Training Content
1. Bockhoff et al. (2022)	Training evaluation survey (Bockhoff et al., 2022) Students help-seeker behavior survey (Bockhoff et al., 2022) Vignette representing a student in crisis, plus 12-item survey (Bockhoff et al., 2022) School staff’s self-efficacy in counseling suicidal students survey (Bockhoff et al., 2022)	QPR
2. Exner-Cortens et al. (2022)	Survey of Knowledge Attitudes (Exner-Cortens et al., 2022) Gatekeeper Behaviors for Suicide Prevention in School Training (Exner-Cortens et al., 2022)	QPR
3. Johnson and Parsons (2012)	Survey designed by researchers	QPR
4. Lamis et al. (2017)	Knowledge Attitudes and Self-efficacy (Lamis et al., 2017)	Making Educators Partners in Suicide Prevention
5. Reis and Cornell (2018)	Student suicide prevention survey (Reis & Cornell, 2018)	QPR
6. Robinson et al. (2016)	Knowledge of Deliberate Self-Harm Questionnaire (Robinson et al., 2016) Attitudes to Children who Self-Harm Scale (Robinson et al., 2016) Attitudes to Suicide Prevention Scale (Appleby et al., 2000) Filmed role play	STORM
7. Shilubane et al. (2015)	5 Focus groups	No training
8. Wyman et al. (2008)	Staff suicide prevention survey (Wyman et al., 2008) Knowledge of QPR (Quinnett, 1999) Appraisals (gatekeeper preparedness, self-evaluation of knowledge, gatekeeper efficacy, gatekeeper reluctance, access to services, gatekeeper behaviors, training) (Wyman et al., 2008) Communication with students (Wyman et al., 2008)	QPR
9. Yamaguchi et al. (2023)	Survey assessing (a) knowledge about suicide, (b) intention to ask about students’ suicidal thoughts/plans and (c) attitudes towards talking to suicidal students (Yamaguchi et al., 2023)	No training

## Data Availability

Data supporting this review are available from the corresponding author on reasonable request, following data sharing agreements.

## Supplementary Materials

The following supporting information can be downloaded at https://www.mdpi.com/article/10.3390/bs16010115/s1, Table S1. Selected Studies—The mental health literacy of secondary school teachers relating to student depression, anxiety, early psychosis or suicide risk.
