# Supplementary material for: Secondary School Teachers’ Disorder-Specific Mental Health Literacy About Depression, Anxiety, Early Psychosis and Suicide Risk: A Scoping Review
Source: Behav Sci (Basel). 2026 Jan 14;16(1):115. doi: 10.3390/bs16010115 (PMC12837168; doi:10.3390/bs16010115)
Supplement: Supplementary file 1 [file behavsci-16-00115-s001.zip › behavsci-4048074-supplementary.pdf]

Table S1 Selected Studies - The mental health literacy of secondary school teachers relating to student depression, anxiety, early psychosis or suicide risk

Studies measuring mental health literacy about depression						
Citation	Purpose	Design and Location	Participants	Mental health literacy training program.	Major Findings	Limitations
1.Arslan & Karabey, 2023	To assess secondary school students and teachers' knowledge, treatment beliefs, help seeking attitudes and stigma towards mental illness (measures mental health literacy about depression, anxiety and schizophrenia).	Quantitative.<br>Survey methodology.<br><br>Measures implemented:<br>one time point.<br><br>Measures used:<br>Self-report.<br>1.Mental Health Literacy Questionnaire (Jorm 1997) - Vignettes presented of depression, anxiety and one of schizophrenia. Participants asked about what they think is wrong, what help they need, and prognosis).<br><br>2.Beliefs Toward Mental Illness Scale Hirai & Clum, 2000) –	Teachers = 241 (female = 54.8%, mean age = 40.1, SD = 8.3).<br><br>Students = 710 (female = 53.7%, mean age = 16.0, SD = 1.4; Yr 9-12).<br><br>Schools = number not reported.	NIL.<br>Current knowledge assessed only.	Low mental health literacy levels found for teachers and students.<br>Teachers correctly recognised student case vignettes with depression (45.5%),<br>Social phobia (29.7%),<br>schizophrenia (47.9%).	Limited generalisation.<br>Self-selection bias.

		designed to assess negative stereotypes of mental illness: dangerousness, social interpersonal skills, incurability.<br><br>Urban.<br>Turkey.				
2.<br>Jorm et al. 2010	To evaluate a cluster randomised control trial of Mental Health First Aid training course for high school teachers (measures mental health literacy of depression, anxiety disorders).	Quantitative.<br>RCT.<br>Intervention – training<br>Control - Waitlist<br>Quasi-experimental design.<br><br>Measures implemented: pre- and post-training and at 6 months follow-up.<br><br>Self-report measures: Designed by researcher.<br>Teachers:<br>1.Knowledge about mental health problems - 21 questions assessing information taught	Teachers = 327 (female = 65.1%)(intervention = 221, control = 106).<br><br>Time working in schools:<br>Less than 3 yrs = 8.6%<br>3-5 yrs = 13.2%<br>6-10 yrs = 12.6%<br>11-15 yrs = 8.9%<br>16-20 yrs = 11.1%<br>>20 yrs = 45.5%<br><br>Students = 1633 (female = 54%) (intervention = 982, control = 651). Yrs 8-10 students, age range 12-15 years.	Youth Mental Health First Aid course:<br>Part 1 – education about mental disorders in adolescents (depression and anxiety disorders, suicidal thoughts and behaviours, non-suicidal self-injury) and applying a mental health action.<br>Part 2 - for teachers responsible for student welfare. Information about first aid approaches for crises requiring a comprehensive response; giving initial help to students who are experiencing a	Mental Health First Aid training significantly increased teachers' knowledge ($p < 0.001$), reduced stigma and increased confidence in providing help. Effect sizes were small-medium. Teachers who completed 2 days training showed greater knowledge gains.<br>Training did not impact teachers helping behaviours for students or colleagues.<br>Most changes sustained 6 months after training.<br>No effects on teachers' individual support towards students with mental health problems.	Blinding of participants not included. Post-test and follow-up questionnaires self-completed by teachers who knew if they had completed training so social desirability bias may exist Statistically significant findings viewed in context of large number of outcome measures and Type 1 errors may exist.

		about depression, anxiety disorders).<br>2. Recognition of depression in a vignette.<br>3. Stigma towards depressed students.<br>4. Beliefs about treatment of depression.<br>5. Confidence in providing help.<br>6. Intentions to help a depressed student.<br>7. Help provided to students.<br>8. First aid to colleagues.<br>9. School practices and policies.<br>10. Teacher psychological distress.<br><br>Students:<br>Administered at pre-test and follow-up.<br>1.Recognition of depression in a vignette.<br>2. Stigma towards a depressed peer.	Schools = 14 schools (intervention = 7, control = 7).	psychotic or an eating disorder or substance misuse.<br>14 hours of face to face training.		

		3. Beliefs in the helpfulness of school staff for a depressed student.<br>4. Help received from school staff.<br>5. Information received from teachers.<br>6. Strengths and Difficulties Questionnaire.<br><br>Urban and rural. Australia.				
3.<br>Miller et al. 2019.	To investigate depression literacy and stigma with teachers and relating to their students (measures mental health literacy of depression).	Quantitative.<br>RCT.<br>Intervention – training<br>Control - Waitlist<br><br>Measures implemented:<br>pre- and post-education intervention.<br><br>Teachers and students completed the same measures.<br><br>Self-report measures:	Teachers = 66 (female = 57.6%; age range 25-64 yrs).<br><br>Years of experience:<br>2-4 yrs = 4.5%<br>5-6 yrs = 3%<br>7-9 yrs = 7.6%<br>>10 yrs =84.8%<br><br>Students = 6679 (age range 14–16 yrs).<br><br>Schools = 54	The Adolescent Depression Awareness Program (ADAP) is a depression literacy program delivered to high school students by teachers.<br><br>Teachers received manualized training. Program included an overview of mood disorders, , and school-based implementation instructions. Teachers given supplemental ADAP educational kits	Teacher literacy was significantly associated with student depression literacy at post-assessment, but not associated with stigma. Teacher stigma was not significantly related to student depression literacy or stigma in the post-assessment.	Small sample size and schools were limited to certain geographic regions, limiting the generalisability of results. Approximately 80% of teachers completed the post-ADAP training survey. Teachers not completing the post-test survey may have had different depression literacy and stigma scores than participating teachers. Self-report survey data may have

		1. The Adolescent Depression Knowledge Questionnaire (ADKQ).<br>2.The Reported and Intended Behaviour Scale (RIBS).<br><br>Urban or rural not reported.<br>USA.		consisting of a teaching manual, Power Point lectures, group activities, handouts, and DVDs.<br>Implemented in 2–3 classes. Teachers completed fidelity assessments.<br>6 hours face to face training.		resulted in social desirability bias.
4.<br>Moor et al. 2007.	To evaluate effectiveness of school-based psychoeducational intervention designed to help teachers recognise symptoms of depression in students (measures mental health literacy of depression).	Quantitative.<br>RCT.<br>Intervention – training<br>Control - Waitlist<br><br>Parental consent required.<br><br>Measures implemented pre and post intervention.<br><br>Self-report measures:<br>Pre-training measures<br><br>Teachers Attitude Questionnaire developed by the researchers.<br>Teachers given class lists of cohort to indicate which	Teachers = 151 (females = 101; intervention = 75, control = 75).<br><br>Students = 1916 (Mean age = 14.5 yrs, range = 13.3 - 16.9 yrs)<br><br>Schools = 8	The teaching package 2 hours face to face in three parts.<br>1. Video of adolescent depression, early detection, role of teachers.<br>2. Series of case vignettes depicting student difficulties most schools would encounter presented to teachers in small discussion groups.<br>3. Discussion of issues specific to local services, and their referral of hypothetical at-risk student.	This training package did not improve ability to recognise depressed students.<br>Teachers in the intervention group recognised 52% of cases before the intervention and 45% afterwards. The control teachers recognised 41% and 43%. No improvement recognising depressed pupils resulted from the training.	Inherent measurement errors in accurate screening was dependant on accurate self-reporting by the adolescent students and disclosure at interview. Teachers used current and previous school term as time frame for consideration (less than 6 months).<br>Students, reported on their recent mood for the screening.

		students they believed were 'possibly/probably' depressed.<br>Students:<br>1.Mood and Feelings Questionnaire (MFQ) .<br>2.Semi-structured clinical interview (K-SADS).<br><br>Urban or rural not stated.<br>United Kingdom				
5. O'Dea et al. 2023	To evaluate the effectiveness of a professional development training program aimed to improve secondary school teachers' confidence, behaviour, knowledge, and attitudes toward student mental health (measures mental health literacy of depression, anxiety).	Quantitative.<br>RCT.<br>Intervention – training<br>Control - Waitlist<br><br>Measures implemented: baseline, post-intervention and 3-month follow-up.<br><br>Self-report measures:<br>1.Confidence in Recognising and Responding to Students' Mental Health Needs (measures mental health literacy of	Teachers/educator = 295 (female = 76.6%, male = 23.3%; Mean age: 40.10 yrs, SD: 10.47 yrs).<br>Participants: Year Advisor or equivalent, Student Coordinator, or Head of Student Wellbeing.<br><br>Educator experience: Mean 13.58 yrs (SD = 9.96) range 0-44 yrs.<br><br>Experience in current role = M 4.5 yrs (SD = 4.42), range 0-28 yrs.	The Black Dog Institute developed the Building Educators' Skills in Adolescent Mental health (BEAM) program. Program targets educators' confidence in recognising and supporting students with mental health problems (depression and anxiety).<br>Online and face to face training conducted over 10 weeks. Unknown hours of training.<br><br>Control – school as usual.	Educators who received the BEAM training program reporting significantly higher levels of confidence at post-intervention and 3-month follow-up, relative to the control. No significant effects found for helping behaviours, stigma, or personal levels of psychological distress.	Study impacted by significant attrition rate (at 3 month follow up 61.2% did not complete). Study relied on self-reported measures to evaluate effectiveness. Lack of standardised instruments to specifically measure teachers' self-efficacy regarding student mental health.

		depression and anxiety).<br>2. Frequency of Helping Behaviours for Student Mental Health.<br>3. Perceived Knowledge and the Perceived Mental .<br>4. Mental Health Literacy.<br>5. Mental Health Stigma.<br>6. 5-item self-report Distress Questionnaire.<br><br>Urban and rural. Australia.	Schools = 73 (22 in rural-regional locations)			
6. Parker et al. 2021	To examine the effectiveness of the Building Educators' skills in Adolescent Mental Health (BEAM) program for improving mental health knowledge in secondary school teachers (measures mental health literacy of depression).	Quantitative. Uncontrolled, single-arm pilot study.<br><br>Measures implemented: baseline, postintervention (6-weeks post baseline) and 3-month follow-up).	Teachers = 70 (female = 70%; mean age 36.5 yrs (SD 9.41), range 24-60 yrs). In the role of Year Advisor or equivalent, Student Coordinator, Head of Year.<br><br>Experience in current role = M = 3.33 yrs (2.97); range 0-15 yrs.	The Black Dog Institute developed a training program delivered over 6 weeks - BEAM program. Combined self-directed content with in-person peer coaching activities and printable resources. 6 hours online training.	The BEAM program was not associated with improvements in mental health knowledge or attitudes. Significant improvements in confidence reported at postintervention and 3-month follow-up. Significant improvements in helping behaviours reported at 3-month follow-up only. Participants reported content easy to understand and relevant, but program	Study impacted by high attrition, for both survey completion and program use, thus limiting conclusions about program effectiveness and acceptability. Only 11 participants completed the program.<br>25% of participants were not confident the program could meet their training needs. Some participants reported that

		Self-reported measures:<br>1.Mental Health Knowledge Schedule (MAKS)<br>2. Depression Stigma Scale.<br>3. Confidence in Helping subscale.<br>4. Help Provided to Students questionnaire.<br>5. Distress Questionnaire-5 (DQ5).<br>6. Perceived Effectiveness. Researcher developed.<br>7. Program Use and Barriers. Researcher developed.<br>8. Program Acceptability and Satisfaction.<br><br>Researcher developed.<br><br>Urban and rural. Australia.	Experience as a secondary school teacher = M 9.62 yrs (6.82); 2-36 yrs.<br><br>Duration of Employment at current school = Mean 6.23 yrs (4.40); 1-23 years.<br><br>Schools = 28 (urban = 30%, regional = 41%, rural = 29%).		completion challenged by lack of time, competing priorities, and forgetfulness.	the skills needed to care for students' mental health could not be taught (or learned) via a self-directed web-based program.
7.	To evaluate teachers' ability to	Quantitative.	Teachers = 32 (female = 68.8%).	Training program focused on education	Before training, majority of teachers could recognise	Small sample size of teachers, based on a

Vieira et al. 2014.	identify and refer students with possible mental health problems, and the effectiveness of a psychoeducational strategy to build capability (measures mental health literacy of depression and psychosis).	Survey including two investigations:<br>1.Longitudinal study with measures obtained before and after training to evaluate effectiveness of mental health training for school teachers.<br>2. Independent case-control study to evaluate teachers' current ability to identify possible mental health problems within their student population.<br><br>Measures implemented: pre and post training.<br><br>Self-report measures:<br>Teacher:<br>1. Questionnaire with six vignettes highlighting behaviours indicating high risk for psychosis, depression, conduct disorder, hyperactivity,	Students = 52 (female = 19.2%; age range 11–17 yrs) (intervention = 26, control = 26). Students were included on teachers' hypothetical lists as possibly having mental health problems.<br><br>School = 1	about types of mental health problems affecting adolescents and impact on school life. Training included differences between normal behaviours and abnormal behaviours that may be warning signs of mental illness. Information about when and where to refer students included. Teachers asked to read vignettes presented and identify whether the student was experiencing a mental health problem and if a need for referral. Teachers not asked to identify the individual conditions.<br>4 hour face to face and online training.	mental health problems in students depicted in vignettes and appropriately refer them. 80.0% of teachers already knew how to correctly identify the vignette of depression and 76.7% for psychosis and to make the appropriate referral before training.<br>Training improved recognition of normal adolescent behaviour only.	representative sample from one public school in Brazil, reducing generalisability.<br>Reduced power within the case-control study as 80.8% of the sample were males. Vignettes did not include anxiety, a prevalent adolescent disorder.

		mania, and normal adolescent behaviour.<br>2.Self-report qualitative evaluation of the training program.<br><br>Students:<br>1.Youth Self-Report (YSR).<br><br>Urban.<br>Brazil.				
8.<br>Wei et al. 2021.	To evaluate the 'Go-To Educator Training' (GTET), targeting educators for improving mental health knowledge and early identification skills (measures mental health literacy of depression, anxiety, early psychosis)	Quantitative.<br>Survey methodology.<br>Pre-test post-test design.<br><br>Measures implemented: pre- and post-training.<br><br>Self-report measures: Designed by the researchers.<br>1.Mental health knowledge and stigma survey (measures knowledge about schizophrenia, depression, bipolar	School staff = 949 (teachers = 493, school mental health professionals = 308, administrators = 110; not stated = 38; female = 78%).<br><br>Schools – not reported.	Six modules designed to be taught in sequence Topics include: basic functions of the brain; different types of mental health problems; best evidence supported treatments; young people's experiences of mental illness; strategies to fight stigma; how to access mental health care, enhancement of mental health self-care. Provides teacher-ready core materials such as lesson plans, classroom	Participants' knowledge improved significantly (large effects) at post-test (M = 20.42, SD = 3.82; 68% correct responses), compared with pre-test (M = 11.66, SD = 4.43; 39% correct responses) $p < .001$. Stigma scores inversely related to increase in knowledge scores, indicating increasing knowledge may be an effective way to reduce stigma.	'Go-To Educators' participants pre-selected by each school's administration, which may introduce bias. Social desirability bias may exist as survey was self-report. Short-term impact of GTET reported so further study necessary to determine if results are maintained over time.

		disorder, anxiety, eating disorders, ADHD, substance abuse).<br><br>Urban or rural not stated.<br>Canada.		activities, print and video resources. 2 day face to face and online training.		
9.<br>Wei & Kutcher, 2014.	To evaluate the effectiveness of mental health training 'Go-to' for educators about early identification of mental disorders, triage and support, and attitudes toward mental illness (measures mental health literacy of depression, anxiety, early psychosis).<br>.	Quantitative. Quasi-experimental design<br><br>Measures implemented: pre- and post-training.<br><br>Measured implemented: Developed by the researchers.<br>1.Knowledge and attitude questionnaire (measures knowledge of schizophrenia, depression, bipolar disorder, anxiety, eating disorders, ADHD, substance abuse).	School staff = 120 (teachers = 70%, counsellors = 17%, administrators = 6%; social workers = 1%, school nurse, health practitioners = 5%; female = 85, male = 34, nonbinary = 1).<br><br>Schools = 40.	'Go-to' Educator Training assumed there were educators with whom students form good relationships and go to for help. Training provided mental health knowledge, identification and support, and strategies for working with mental health service providers, parents, and families. Participants were joined by local mental health providers to establish collaboration networks to facilitate appropriate care.<br>1 day online training.	Participants mean scores on mental health competencies changed from 12 (40%) (*SD* = 4.3) to 21 (70%) (*SD* = 3.3) post training, $p < .0001$.<br>Participant attitude mean scores improved from 49.9 (*SD* = 4.6) pretraining, to 51.5 (*SD* = 4.2), $p < .0001$.<br>Prior to the training, group correctly answered a mean (M) score of 12 of 30 (Standard Deviation [SD] = 4.3) for mental health knowledge questions, improving to a mean group score of 21 (SD = 3.3) following the training, a statistically significant change. Training significantly improved educators' knowledge to	School principals rather than students identified 'go-to' educators that may have biased results. Social desirability bias may exist. No follow up measure to evaluate longer term impact of the training. A preliminary study. Randomised controlled trial needed to evaluate robustness for wider generalisability.

		Urban or rural Canada.			identify adolescents with mental disorders and link them with appropriate services.	

Studies measuring mental health literacy about anxiety						
Citation	Purpose	Design and Location	Participants	Mental health literacy training program.	Major Findings	Limitations
1.Arslan & Karabey, 2023	To assess secondary school students and teachers' knowledge, treatment beliefs, help seeking attitudes and stigma towards mental illness (measures mental health literacy about depression, anxiety and schizophrenia).	Quantitative.<br>Survey methodology.<br><br>Measures implemented:<br>one time point.<br><br>Measures used:<br>Self-report.<br>1.Mental Health Literacy Questionnaire (Jorm 1997) - Vignettes presented of depression, anxiety and one of schizophrenia. Participants asked about what they think is wrong, what help they need, and prognosis).	Teachers = 241 (female = 54.8%, mean age = 40.1, SD = 8.3).<br><br>Students = 710 (female = 53.7%, mean age = 16.0, SD = 1.4; Yr 9-12).<br><br>Schools = number not reported.	NIL.<br>Current knowledge assessed only.	Low mental health literacy levels found for teachers and students.<br>Teachers correctly recognised student case vignettes with depression (45.5%),<br>Social phobia (29.7%),<br>schizophrenia (47.9%).	Limited generalisation.<br>Self-selection bias.

		2.Beliefs Toward Mental Illness Scale Hirai & Clum, 2000) – designed to assess negative stereotypes of mental illness: dangerousness, social interpersonal skills, incurability.<br><br>Urban.<br>Turkey.				
2.<br>Jorm et al. 2010	To evaluate a cluster randomised control trial of Mental Health First Aid training course for high school teachers (measures mental health literacy of depression, anxiety disorders).	Quantitative.<br>RCT.<br>Intervention – training<br>Control - Waitlist<br>Quasi-experimental design.<br><br>Measures implemented: pre- and post-training and at 6 months follow-up.<br><br>Self-report measures: Designed by researcher.<br>Teachers:<br>1.Knowledge about mental health	Teachers = 327 (female = 65.1%)(intervention = 221, control = 106).<br><br>Time working in schools:<br>Less than 3 yrs = 8.6%<br>3-5 yrs = 13.2%<br>6-10 yrs = 12.6%<br>11-15 yrs = 8.9%<br>16-20 yrs = 11.1%<br>>20 yrs = 45.5%<br><br>Students = 1633 (female = 54%) (intervention = 982, control = 651). Yrs 8-10 students, age range 12-15 years.	Youth Mental Health First Aid course:<br>Part 1 – education about mental disorders in adolescents (depression and anxiety disorders, suicidal thoughts and behaviours, non-suicidal self-injury) and applying a mental health action.<br>Part 2 - for teachers responsible for student welfare. Information about first aid approaches for crises requiring a comprehensive	Mental Health First Aid training significantly increased teachers’ knowledge ($p < 0.001$), reduced stigma and increased confidence in providing help. Effect sizes were small-medium. Teachers who completed 2 days training showed greater knowledge gains.<br>Training did not impact teachers helping behaviours for students or colleagues.<br>Most changes sustained 6 months after training.<br>No effects on teachers' individual support towards	Blinding of participants not included. Post-test and follow-up questionnaires self-completed by teachers who knew if they had completed training so social desirability bias may exist Statistically significant findings viewed in context of large number of outcome measures and Type 1 errors may exist.

		problems - 21 questions assessing information taught about depression, anxiety disorders.<br>2. Recognition of depression in a vignette.<br>3. Stigma towards depressed students.<br>4. Beliefs about treatment of depression.<br>5. Confidence in providing help.<br>6. Intentions to help a depressed student.<br>7. Help provided to students.<br>8. First aid to colleagues.<br>9. School practices and policies.<br>10. Teacher psychological distress.<br><br>Students:<br>Administered at pre-test and follow-up.	Schools = 14 schools (intervention = 7, control = 7).	response; giving initial help to students who are experiencing a psychotic or an eating disorder or substance misuse.<br>14 hours of face to face training.	students with mental health problems.	

		1.Recognition of depression in a vignette.<br>2. Stigma towards a depressed peer.<br>3. Beliefs in the helpfulness of school staff for a depressed student.<br>4. Help received from school staff.<br>5. Information received from teachers.<br>6. Strengths and Difficulties Questionnaire.<br><br>Urban and rural. Australia.				
3. O'Dea et al. 2023.	To evaluate the effectiveness of a professional development training program aimed to improve secondary school teachers' confidence, behaviour, knowledge, and	Quantitative.<br>RCT.<br>Intervention – training<br>Control - Waitlist<br><br>Measures implemented: baseline, post-intervention and 3-month follow-up.<br><br>Self-report measures:	Teachers/educator = 295 (female = 76.6%, male = 23.3%; Mean age: 40.10 yrs, SD: 10.47 yrs).<br>Participants: Year Advisor or equivalent, Student Coordinator, or Head of Student Wellbeing.<br><br>Educator experience:	The Black Dog Institute developed the Building Educators' Skills in Adolescent Mental health (BEAM) program. Program targets educators' confidence in recognising and supporting students with mental health problems (depression and anxiety).	Educators who received the BEAM training program reporting significantly higher levels of confidence at post-intervention and 3-month follow-up, relative to the control. No significant effects found for helping behaviours, stigma, or personal levels of psychological distress.	Study impacted by significant attrition rate (at 3 month follow up 61.2% did not complete). Study relied on self-reported measures to evaluate effectiveness. Lack of standardised instruments to specifically measure teachers' self-efficacy regarding student mental health.

	attitudes toward student mental health (measures mental health literacy of depression, anxiety).	1.<br>Confidence in Recognising and Responding to Students' Mental Health Needs (measures mental health literacy of depression, anxiety).<br>2. Frequency of Helping Behaviours for Student Mental Health<br>.<br>3. Perceived Knowledge and the Perceived Mental .<br>4. Mental Health Literacy .<br>5. Mental Health Stigma .<br>6. 5-item self-report Distress Questionnaire<br>.<br><br>Urban and rural. Australia.	Mean 13.58 yrs (SD = 9.96) range 0-44 yrs.<br><br>Experience in current role = M 4.5 yrs (SD = 4.42), range 0-28 yrs.<br><br>Schools = 73 (22 in rural-regional locations)	Online and face to face training conducted over 10 weeks. Unknown hours of training.<br><br>Control – school as usual.		
4.<br>Wei et al. 2021.	To evaluate the 'Go-To Educator Training' (GTET), targeting educators for improving mental health	Quantitative.<br>Survey methodology.<br>Pre-test post-test design.	School staff = 949 (teachers = 493, school mental health professionals = 308, administrators = 110; not stated = 38; female = 78%).	Six modules designed to be taught in sequence Topics include: basic functions of the brain; different types of mental health	Participants' knowledge improved significantly and substantially (large effects) at post-test (M = 20.42, SD = 3.82; 68% correct responses),compared with	'Go-To Educators' participants pre-selected by each school's administration, which may introduce bias. Social desirability bias may exist as survey was self-report.

	knowledge and early identification skills (measures mental health literacy of depression, anxiety, early psychosis).	Measures implemented: pre- and post-training.<br><br>Self-report measures: Designed by the researchers.<br>1.Mental health knowledge and stigma survey (measures knowledge about schizophrenia, depression, bipolar disorder, anxiety, eating disorders, ADHD, substance abuse).<br><br>Urban or rural not stated.<br>Canada.	Schools – not reported.	problems; best evidence supported treatments; young people's experiences of mental illness; strategies to fight stigma; how to access mental health care, enhancement of mental health self-care. Provides teacher-ready core materials such as lesson plans, classroom activities, print and video resources. 2 day face to face and online training.	pre-test (M = 11.66, SD = 4.43; 39% correct responses) p < .001. Stigma scores inversely related to increase in knowledge scores, indicating increasing knowledge may be an effective way to reduce stigma.	Short-term impact of GTET reported so further study necessary to determine if results are maintained over time.
5.<br>Wei & Kutcher, 2014.	To evaluate the effectiveness of mental health training 'Go-to' for educators about early identification of mental disorders, triage and support, and attitudes	Quantitative. Quasi-experimental design<br><br>Measures implemented: pre- and post-training.	School staff = 120 (teachers = 70%, counsellors = 17%, administrators = 6%; social workers = 1%, school nurse, health practitioners = 5%; female = 85, male = 34, nonbinary = 1).	'Go-to' Educator Training assumed there were educators with whom students form good relationships and go to for help. Training provided mental health knowledge, identification and	Participants mean scores on mental health competencies changed from 12 (40%) (*SD* = 4.3) to 21 (70%) (*SD* = 3.3) post training, $p < .0001$. Participant attitude mean scores improved from 49.9	School principals rather than students identified 'go-to' educators that may have biased results. Social desirability bias may exist. No follow up measure to evaluate longer term impact of the training. A preliminary

	toward mental illness (measures mental health literacy of depression, anxiety, early psychosis).	Measured implemented: Developed by the researchers. 1.Knowledge and attitude questionnaire (schizophrenia, depression, bipolar disorder, anxiety, eating disorders, ADHD, substance abuse).<br><br>Urban or rural Canada.	Schools = 40.	support, and strategies for working with mental health service providers, parents, and families. Participants were joined by local mental health providers to establish collaboration networks to facilitate appropriate care.<br>1 day online training.	(*SD* = 4.6) pretraining, to 51.5 (*SD* = 4.2), *p* < .0001.<br>Prior to the training, group correctly answered a mean (M) score of 12 of 30 (Standard Deviation [SD] = 4.3) for mental health knowledge questions, improving to a mean group score of 21 (SD = 3.3) following the training, a statistically significant change. Training significantly improved educators' knowledge to identify adolescents with mental disorders and link them with appropriate services.	study. Randomised controlled trial needed to evaluate robustness for wider generalisability.

Studies measuring mental health literacy about early psychosis

Citation	Purpose	Design and Location	Participants	Mental health literacy training program.	Major Findings	Limitations
1.Arslan & Karabey, 2023	To assess secondary school students and teachers' knowledge, treatment beliefs,	Quantitative.<br>Survey methodology.<br><br>Measures implemented:	Teachers = 241 (female = 54.8%, mean age = 40.1, SD = 8.3).	NIL.<br>Current knowledge assessed only.	Low mental health literacy levels found for teachers and students.<br>Teachers correctly recognised student case vignettes with	Limited generalisation. self-selection bias.

	help seeking attitudes and stigma towards mental illness (measures mental health literacy about depression and schizophrenia).	one time point.<br><br>Measures used:<br>Self-report.<br>1.Mental Health Literacy Questionnaire (Jorm 1997) - Vignette presented of depression and one of schizophrenia. Participants asked about what they think is wrong, what help they need, and prognosis).<br><br>2.Beliefs Toward Mental Illness Scale Hirai & Clum, 2000) – designed to assess negative stereotypes of mental illness: dangerousness, social interpersonal skills, incurability.<br><br>Urban.<br>Turkey.	Students = 710 (female = 53.7%, mean age = 16.0, SD = 1.4; Yr 9-12).<br><br>Schools = number not reported.		depression (45.5%), schizophrenia (47.9%).	
2.<br>Jorm et al. 2010	To evaluate a cluster randomised control trial of Mental Health First Aid training course	Quantitative.<br>RCT.<br>Intervention – training<br>Control - Waitlist	Teachers = 327 (female = 65.1%)(intervention = 221, control = 106).	Youth Mental Health First Aid course:<br>Part 1 – education about mental disorders in adolescents	Mental Health First Aid training significantly increased teachers’ knowledge ($p < 0.001$), reduced stigma and increased confidence in	Blinding of participants not included. Post-test and follow-up questionnaires self-completed by teachers

	for high school teachers (measures mental health literacy of depression, anxiety disorders).	Quasi-experimental design.<br><br>Measures implemented: pre- and post-training and at 6 months follow-up.<br><br>Self-report measures: Designed by researcher.<br>Teachers:<br>1.Knowledge about mental health problems - 21 questions assessing information taught about depression, anxiety disorders.<br>2. Recognition of depression in a vignette.<br>3. Stigma towards depressed students.<br>4. Beliefs about treatment of depression.<br>5. Confidence in providing help.<br>6. Intentions to help a depressed student.	Time working in schools:<br>Less than 3 yrs = 8.6%<br>3-5 yrs = 13.2%<br>6-10 yrs = 12.6%<br>11-15 yrs = 8.9%<br>16-20 yrs = 11.1%<br>>20 yrs = 45.5%<br><br>Students = 1633 (female = 54%) (intervention = 982, control = 651). Yrs 8-10 students, age range 12-15 years.<br><br>Schools = 14 schools (intervention = 7, control = 7).	(depression and anxiety disorders, suicidal thoughts and behaviours, non-suicidal self-injury) and applying a mental health action.<br>Part 2 - for teachers responsible for student welfare. Information about first aid approaches for crises requiring a comprehensive response; giving initial help to students who are experiencing a psychotic or an eating disorder or substance misuse.<br>14 hours of face to face training.	providing help. Effect sizes were small-medium. Teachers who completed 2 days training showed greater knowledge gains.<br>Training did not impact teachers helping behaviours for students or colleagues.<br>Most changes sustained 6 months after training.<br>No effects on teachers' individual support towards students with mental health problems.	who knew if they had completed training so social desirability bias may exist Statistically significant findings viewed in context of large number of outcome measures and Type 1 errors may exist.

		7. Help provided to students.<br>8. First aid to colleagues.<br>9. School practices and policies.<br>10. Teacher psychological distress.<br><br>Students: Administered at pre-test and follow-up.<br>1.Recognition of depression in a vignette.<br>2. Stigma towards a depressed peer.<br>3. Beliefs in the helpfulness of school staff for a depressed student.<br>4. Help received from school staff.<br>5. Information received from teachers.<br>6. Strengths and Difficulties Questionnaire.<br><br>Urban and rural.				

		Australia.				
3.<br>Vieira et al. 2014.	To evaluate teachers' ability to identify and refer students with possible mental health problems, and the effectiveness of a psychoeducational strategy to build capability (measures mental health literacy of depression and psychosis).	Quantitative.<br>Survey including two investigations:<br>1.Longitudinal study with measures obtained before and after training to evaluate effectiveness of mental health training for schoolteachers.<br>2. Independent case-control study to evaluate teachers' current ability to identify possible mental health problems within their student population.<br><br>Measures implemented: pre and post training.<br><br>Self-report measures:<br>Teacher:<br>1. Questionnaire with six vignettes highlighting behaviours indicating high risk for psychosis,	Teachers = 32 (female = 68.8%).<br><br>Students = 52 (female = 19.2%; age range 11–17 yrs) (intervention = 26, control = 26). Students were included on teachers' hypothetical lists as possibly having mental health problems.<br><br>School = 1	Training program focused on education about types of mental health problems affecting adolescents and impact on school life. Training included differences between normal behaviours and abnormal behaviours that may be warning signs of mental illness. Information about when and where to refer students included. Teachers asked to read vignettes presented and identify whether the student was experiencing a mental health problem and if a need for referral. Teachers not asked to identify the individual conditions.<br>4 hour face to face and online training.	Before training, majority of teachers could recognise mental health problems in students depicted in vignettes and appropriately refer them. 80.0% of teachers already knew how to correctly identify the vignette of depression and 76.7% for psychosis and to make the appropriate referral before training.<br>Training improved recognition of normal adolescent behaviour only.	Small sample size of teachers, based on a representative sample from one public school in Brazil, reducing generalisability.<br>Reduced power within the case-control study as 80.8% of the sample were males. Vignettes did not include anxiety, a prevalent adolescent disorder.

		depression, conduct disorder, hyperactivity, mania, and normal adolescent behaviour.<br>2.Self-report qualitative evaluation of the training program.<br><br>Students:<br>1.Youth Self-Report (YSR).<br><br>Urban.<br>Brazil.				
4.<br>Wei et al. 2021.	To evaluate the 'Go-To Educator Training' (GTET), targeting educators for improving mental health knowledge and early identification skills (measures mental health literacy of depression, anxiety, early psychosis).	Quantitative.<br>Survey methodology.<br>Pre-test post-test design.<br><br>Measures implemented: pre- and post-training.<br><br>Self-report measures:<br>Designed by the researchers.<br>1.Mental health knowledge and stigma survey (measures knowledge about	School staff = 949 (teachers = 493, school mental health professionals = 308, administrators = 110; not stated = 38; female = 78%).<br><br>Schools – not reported.	Six modules designed to be taught in sequence Topics include: basic functions of the brain; different types of mental health problems; best evidence supported treatments; young people's experiences of mental illness; strategies to fight stigma; how to access mental health care, enhancement of mental health self-care. Provides teacher-ready	Participants' knowledge improved significantly and substantially (large effects) at post-test ($M = 20.42$, $SD = 3.82$; 68% correct responses),compared with pre-test ($M = 11.66$, $SD = 4.43$; 39% correct responses) $p < .001$. Stigma scores inversely related to increase in knowledge scores, indicating increasing knowledge may be an effective way to reduce stigma.	'Go-To Educators' participants pre-selected by each school's administration, which may introduce bias. Social desirability bias may exist as survey was self-report. Short-term impact of GTET reported so further study necessary to determine if results are maintained over time.

		depression, anxiety, early psychosis, PTSD, ADHD).<br><br>Urban or rural not stated.<br>Canada.		core materials such as lesson plans, classroom activities, print and video resources. 2 day face to face and online training.		
5.<br>Wei & Kutcher, 2014.	To evaluate the effectiveness of mental health training 'Go-to' for educators about early identification of mental disorders, triage and support, and attitudes toward mental illness (measures mental health literacy of depression, anxiety, early psychosis).<br>.	Quantitative. Quasi-experimental design<br><br>Measures implemented: pre- and post-training.<br><br>Measured implemented: Developed by the researchers.<br>1.Knowledge and attitude questionnaire (schizophrenia, depression, bipolar disorder, anxiety, eating disorders, ADHD, substance abuse).<br><br>Urban or rural Canada.	School staff = 120 (teachers = 70%, counsellors = 17%, administrators = 6%; social workers = 1%, school nurse, health practitioners = 5%; female = 85, male = 34, nonbinary = 1).<br><br>Schools = 40.	'Go-to' Educator Training assumed there were educators with whom students form good relationships and go to for help. Training provided mental health knowledge, identification and support, and strategies for working with mental health service providers, parents, and families. Participants were joined by local mental health providers to establish collaboration networks to facilitate appropriate care.<br>1 day online training.	Participants mean scores on mental health competencies changed from 12 (40%) (SD = 4.3) to 21 (70%) (SD = 3.3) post training, $p < .0001$.<br>Participant attitude mean scores improved from 49.9 (SD = 4.6) pretraining, to 51.5 (SD = 4.2), $p < .0001$.<br>Prior to the training, group correctly answered a mean (M) score of 12 of 30 (Standard Deviation [SD] = 4.3) for mental health knowledge questions, improving to a mean group score of 21 (SD = 3.3) following the training, a statistically significant change. Training significantly improved educators' knowledge to identify adolescents with mental disorders and link	School principals rather than students identified 'go-to' educators that may have biased results. Social desirability bias may exist. No follow up measure to evaluate longer term impact of the training. A preliminary study. Randomised controlled trial needed to evaluate robustness for wider generalisability.

					them with appropriate services.	

Studies measuring mental health literacy about suicide risk						
Citation	Purpose	Design and Location	Participants	Mental health literacy training program.	Major Findings	Limitations
1. Bockhoff et al. 2022	To evaluate a suicide prevention gatekeeper training program for teachers and school social workers.	Quantitative.<br>A two-factor experimental design.<br><br>Four study conditions:<br>1. gatekeeper training for school staff,<br>2. workshop for students,<br>3. training programs for both,<br>4. control group.<br><br>Measures implemented: pre-test, post-test, and follow-up, approximately 3 months training.<br><br>Self-report measures: Researcher developed.<br>1.Training evaluation survey.	Teachers = 129 (86% of sample) (female = 77%).<br>School social workers = 18 (12%).<br><br>Students = 200 (females = 79%).<br>Secondary school, year level not specified.<br><br>Schools = 12	Condition 1: Gatekeeper training based on Quinnett's (2012) Question, Persuade and Refer program, including interviewing techniques, action plans, development of skills in recognising students at suicide risk.<br>12 hours of face to face training.<br><br>Condition 2:<br>The student workshop on psychoeducation focused on suicidality, coping strategies and Acceptance and Commitment principles.<br>4 hours training.<br><br>Control: school as usual.	Participants who completed gatekeeper training improved significantly in knowledge, and QPR skills in comparison to the control group.	The small sample size reduces generalisability. .<br>Predominantly female sample prevents conclusions regarding gender differences.<br>Results may have been biased by the self-selection of participants.

		2. Students help-seeker behaviour.<br>3. Vignette representing a student in crisis plus 12-item survey.<br>4. School staff's self-efficacy in counselling students in need survey.<br><br>Urban.<br>Germany.				
2.<br>Exner-Cortens et al. 2022.	To evaluate a mixed method study focused on natural leader training to support the implementation of a Question, Persuade and Refer (QPR) Gatekeeper Suicide Prevention Program in schools.	Mixed methods.<br>Intervention = QPR® + Natural Leader (NL) training,<br>Control = QPR® training only.<br><br>Measures implemented:<br>pre training, one week post training, 2 months post training.<br><br>Self-report measures:<br>1. Survey of Knowledge Attitudes, developed by researchers.	Teachers = 26 (female = 76.9%; age range 20-60 yrs).<br><br>Years of experience:<br>1-5 yrs = 15.4%<br>6-10 yrs = 19.2%<br>10-15 yrs = 23.1%<br><br>Teaching:<br>Junior School = 30.8%<br>Secondary School = 69.2%.<br><br>Schools = number no reported.	The gatekeeper training was online QPR® training .<br>60 minutes online training.<br><br>Natural Leader (NL) Training consisted of videos (12 hours) and three x 60-minute sessions via Zoom.<br>Activities included (a) the potential impact of stigma on teacher responses to students in distress , (b) role-playing the QPR® technique and (c) planning to support suicide prevention in their school.	Teachers and staff who participated in QPR® training reported significant changes on the gatekeeper evaluation scale, preparedness to serve as gatekeepers, and knowledge about role.<br>Training increased participant comfort and confidence as a gatekeeper.<br>The natural leader training was well-received, and roleplays increased confidence.	Research interrupted by the Covid-19 pandemic.<br>Small sample size.

		2. Gatekeeper Behaviours for Suicide Prevention in School Training.<br>3. Feedback Form.<br>4.Interviews/<br>Focus Groups.<br><br>Urban and rural.<br>Canada.				
3.<br>Johnson & Parsons, 2012	To evaluate a gatekeeper suicide prevention program for teachers in middle and high students.	Quantitative.<br>Survey methodology.<br><br>Measured implemented: pre and post training. Monthly e-mail for 3-month following the training about implementation of Question, Persuade and Refer use.<br><br>Measures used:<br>9 item survey designed by researchers.<br><br>Urban or rural not stated.<br>USA.	Teachers = 36, middle and high school.<br><br>Schools = 1	QPR Gatekeeper Suicide Prevention<br>Program training; online, evidence-based suicide warning signs, and working with potentially suicidal students. Role-plays of scenarios about asking about suicide, facilitating a student to accept help, and making referrals to the school counsellor.<br>90 min face to face training.	QPR Training significantly increased knowledge in identifying risk factors, responses to suicidal students, knowledge of community resources available.<br>One staff member from 36 reported using the QPR with a suicidal student within 3 months post training.	Questionnaires were self-report so self-selection and social desirability bias may exist.
4.	To evaluate 'Act on FACTS: Making	Quantitative.<br>Survey methodology.	School personnel = 700 (teachers = 620,	Act on FACTS: Making Educators Partners in	MEP increased suicide knowledge, attitudes, and self-	No control group. Limited generalisability, self-

Lamis, et al. 2017	Educators Partners in Youth Suicide Prevention (MEP), an online training program designed to enhance knowledge of suicide risk factors, warning signs, attitudes and self-efficacy.	Measures implemented: pre and post training.<br><br>Self-report measures: Developed by the researcher .<br>1.Knowledge Attitudes and Self-efficacy<br>2.Satisfaction with training.<br><br>Urban or rural not stated.<br>USA.	administrators = 35, teachers' aides = 26, counsellors = 19) (female = 79.6%; Mean age 40.24 yrs, SD = 12.03 yrs).<br><br>Schools = not reported.	Youth Suicide Prevention (MEP), an online training program increasing awareness of youth suicide and recognition of warning signs, improving confidence to refer to services. Online training completed at own pace. Hours required not reported.	efficacy. Moderating effects of change in self-efficacy, but not suicide knowledge or attitudes. Teachers demonstrated significantly larger increases in self-efficacy/confidence compared with administrators.	report data may result in social desirability bias.
5.<br>Reis & Cornell, 2018.	To investigate the effects of gatekeeper suicide prevention training for counsellors and teachers.	Quantitative.<br>Quasi-experimental design.<br>Training group (QPR) versus control group.<br><br>Implemented 4-5 months after training.<br><br>Self-reported measures;<br>1.Student suicide prevention survey.	Training<br>Teachers = 165<br>Counsellors = 73<br><br>Worked in primary school = 26; secondary school = 205; not reported = 7.<br><br>Control<br>Teachers = 98<br>Counsellors = 74	Question, Persuade, and Refer (QPR) training teaches participants to be suspicious for suicidality in people exhibiting warning signs, actively question individuals they suspect might be suicidal, strategies to persuade the suicidal person to take positive, life-saving action in accepting professional help, and	School staff attending QPR training demonstrated significantly greater knowledge of suicide risk factors and reported more active involvement, more confidence, and questioned a lower number of potentially suicidal students than control group $p < 0.001$ (73 counsellors and 165 teachers). They demonstrated greater knowledge of suicide risk	A quasi-experimental design so is not possible to conclude that group differences were caused by the training program. Training participants either volunteered or were sent to training by their supervisors. The control group was selected by contacting school personnel in localities where training had not been offered so

		Designed the researchers.<br><br>Urban and rural.<br>USA.	Worked in primary school = 57 Secondary school = 111; not reported = 4<br><br>Schools<br>Primary = 50<br>Secondary = 140	referring the student for treatment.<br>1 day online training.	factors and reported making more no-harm contracts than controls (74 counsellors and 98 teachers). School counsellors had higher suicide knowledge scores than teachers. Training had no impact increasing staff asking students about suicide, suggesting staff reluctance to question students about suicide is substantial, and might not be easily overcome by training.	cannot rule out preexisting differences between trainees and controls.
6. Robinson et al. 2016.	To examine the impact of delivering an evidence based gatekeeper training package for suicide prevention (STORM®) in an Australian school setting.	Quantitative.<br>Pre-test post-test design.<br><br>Measures implemented:<br>pre training, immediately after training, 8 weeks after training.<br><br>Self-report measures:<br>1.Knowledge of Deliberate Self Harm Questionnaire.	School staff = 84 (teachers = 25, welfare coordinators = 8, psychologists = 15, nurses = 11, principals = 1, teachers assistant = 1; female = 64).<br><br>Schools = 41	STORM is in-depth self-harm mitigation and suicide prevention and postvention training focusing on skill development. It uses filmed role play, lectures, practical demonstrations, skills practice,<br>reflection and feedback. Comprises 4 modules: assessment of suicide risk, suicide safety planning, self-injury	Participants demonstrated increased knowledge, confidence, and skills following the training.	Repeated measures design limited ability to ascertain whether knowledge and confidence changed. No control group for comparison. No conclusions can be drawn about the filmed role plays because only focused on suicide risk assessment immediately post training. Changes sustained over time are unknown.

		Developed by the researchers.<br>2. Attitudes to Children who Self Harm Scale. Developed by the researchers.<br>3. Attitudes to Suicide Prevention Scale.<br>Filmed roles plays.<br><br>Urban<br>Australia		mitigation, and suicide postvention.<br>2 days face to face training.		Sample was randomly selected from the same area.
7. Shilubane et al. 2015.	To assess secondary teachers knowledge of identifying warning signs of suicidal behaviour, the type of information they give to students after a suicide of a classmate, and their views and training needs on the prevention of suicidal behaviour in students.	Qualitative.<br>5 Focus groups.<br><br>Rural.<br>South Africa.	Teachers = 50 (27 females).<br>All had experienced a student die by suicide.<br><br>Schools = 5	No training provided.	Teachers reported a lack knowledge about the warning signs of suicidal students. They did not know how to support students in the event of attempted or completed suicide of another student. Teachers reported no services available at schools to assist suicide survivors (teachers, students, and administration staff) following a student's suicide.	Limited generalisability due to small sample and specific geographic location. Self-selection and response bias may be present.
8. Wyman et al. 2008.	To assess impact of a suicide prevention gatekeeper training	Quantitative.<br>RCT.	School staff = 342 (teachers = 60%, administrators = 12%,	QPR Training covers: rates of youth suicide, warning signs and risk	Appraisal scores increased most for staff with lowest baseline appraisals, and	Only one-third of school staff agreed to participate. Self-selection

	program (Question, Persuade, Refer (QPR) on school staff's knowledge, and identification of students.	Intervention QPR training, Wait list control.<br><br>Randomisation occurred at a school level.<br><br>Measures implemented: pre training, 1 year follow up.<br><br>Self-report measures: Staff Suicide: 1. Staff suicide prevention survey.<br>2.Knowledge of QPR.<br>3.Appraisals (Gatekeeper preparedness, self-evaluation of knowledge, gatekeeper efficacy, gatekeeper reluctance, access to services, gatekeeper behaviours, of training). Designed by researchers.	health/social service staff = 8%, support staff = 20%; female = 81.9%) (intervention = 166, control = 176).<br><br>Students = 2,059<br>8th and 10th grade students completed annual school survey including questions about suicidal ideation/behaviour.<br><br>Schools = 32 (16 training, 16 wait list control).	factors for suicide, procedures for asking a student about suicide, persuading a student to get help, and referring a student for help.<br>90 minutes of face to face training.	suicide identification behaviours increased most for staff already communicating with students about suicide and distress. Increased knowledge did not increase suicide risk identification. Training increased participants' accuracy identifying warning signs and risk factors for youth suicide. QPR did not change staff communication with students about emotional distress.	bias may be present as only those staff interested in student suicide risk may have participated limiting generalisation of results. Reliance on staff self-reports. 77.4% of the sample completed the 1 year follow up.

		3.Communication with students. Designed by researchers.<br><br>Students<br>1.Student Survey.<br><br>Urban and rural.<br>USA.				
9. Yamaguchi et al. 2023.	To investigate suicide literacy in school teachers.	Quantitative.<br>Survey methodology.<br><br>Implemented at one time point.<br><br>Measure used:<br>Designed the researchers:<br>A self-administered survey assessing (a) knowledge about suicide, (b) intention to ask about students' suicidal thoughts/plans, and (c) attitudes towards talking to students with mental health problems.<br><br>Urban or rural not stated.	Teachers = 857 (female = 43.5%, male = 56.4%)<br><br>Schools = 48 (primary = 26.1%, secondary = 73.6%).	No training provided.	55% of teachers knew suicide was a leading cause of adolescent deaths.<br>56.2% knew asking about suicidality was needed.<br>Half the teachers intended to ask students about their suicidal thoughts (50.2%).<br>90.4% agreed that talking to students with mental health problems was a teacher's responsibility.<br>Intention to ask about students' suicidal thoughts/plans were higher in teachers in their 20s (vs. 40s–60s) and working at secondary schools (vs. primary schools).	Participants were from a single area in Japan therefore generalising results to other populations limited. Suicide literacy in participating teachers might differ from those who did not participate. The survey was newly developed and tailored to assess suicide literacy in Japanese teachers. Self-report survey data may have resulted in social desirability bias.

		Japan.				
